# Genetic and environmental influences on the stability of psychotic experiences and negative symptoms in adolescence

**DOI:** 10.1111/jcpp.13045

**Published:** 2019-04-07

**Authors:** Laura Havers, Mark J. Taylor, Angelica Ronald

**Affiliations:** ^1^ Department of Psychological Sciences Birkbeck, University of London London UK; ^2^ Department of Medical Epidemiology & Biostatistics Karolinska Institutet Stockholm Sweden

**Keywords:** Adolescence, aetiology, development, mental health, psychosis

## Abstract

**Background:**

Psychotic experiences (PEs) such as paranoia and hallucinations, and negative symptoms (NS) such as anhedonia and flat affect are common in adolescence. Psychotic experiences and negative symptoms (PENS) increase risk for later psychiatric outcomes, particularly when they persist. The extent to which genetic and environmental influences contribute to the stability of PENS in mid‐to‐late adolescence is unknown.

**Methods:**

Using the Specific Psychotic Experiences Questionnaire (SPEQ) twice across ~9 months in adolescence, *N *=* *1,448 twin pairs [*M *=* *16.32 (0.68)] reported experiences of paranoia, hallucinations, cognitive disorganization, grandiosity and anhedonia, and their parents reported on a range of NS. Individuals were split into low‐scoring, decreasing, increasing and persistent groups for each subscale. Frequencies and mean differences in distress, depression traits and emotional problems were investigated across groups. Longitudinal structural equation modelling was used to estimate the aetiological components underlying the stability of PENS.

**Results:**

Phenotypic stability was moderate for all PENS (*r *=* *.59–.69). Persistent PENS across 9 months were associated with greater levels of distress (*V* = 0.15–0.46, for PEs only), depression traits (*d *=* *0.47–1.67, except grandiosity) and emotional problems (*d *=* *0.47–1.47, except grandiosity and anhedonia) at baseline compared to groups with transitory or low levels of PENS. At both ages PENS were heritable and influenced by shared and nonshared environment. Genetic influences contributed 38%–62% and shared environment contributed 13%–33% to the stability of PENS. Nonshared environment contributed 34%–41% (12% for parent‐rated NS). There was strong overlap of genetic and shared environmental influences across time, and lower overlap for nonshared environment. Imperfect stability of PENS was at least partly due to nonshared environmental influences.

**Conclusions:**

When adolescent PENS persist over time, they are often characterized by more distress, and higher levels of other psychopathology. Both genetic and environmental effects influence stability of PENS.

## Introduction

Experiences such as paranoia, hallucinations, anhedonia and behaviours such as flat affect are reported in childhood and adolescence, and in general population as well as clinical samples (McGrath et al., 2015; Peters et al., 2016; Wong, Freeman, & Hughes, 2014). These experiences and behaviours are grouped together in the study of psychotic or psychotic‐like experiences (PEs), and negative symptoms (NS), because in their extreme they are characteristic of psychotic illnesses. Psychotic experiences and negative symptoms (PENS) show considerable variability in the general population and typically show a positively skewed distribution (e.g., Bebbington et al., 2013; Ronald et al., 2014).

Epidemiological findings suggest that PEs are common (McGrath et al., 2015), associated with earlier childhood behaviour problems (Shakoor, McGuire, Cardno, Freeman, & Ronald, 2018) and that they are cross‐sectionally less prevalent with increasing age (Kelleher, Connor et al., 2012; McGrath et al., 2015). For the majority of people, PEs generally abate (Linscott & van Os, 2013), showing mean‐level decline over time (Dominguez, Wichers, Lieb, Wittchen, & van Os, 2011; Mackie, Castellanos‐Ryan, & Conrod, 2011; Rössler et al., 2007). Some PEs may thus be part of typical behavioural variation (Hanssen, Bak, Bijl, Vollebergh, & van Os, 2005; Van Os, Linscott, Myin‐Germeys, Delespaul, & Krabbendam, 2009; Wong & Raine, 2018; Wong et al., 2014). Longitudinal studies show that child and adolescent PEs are associated with increased odds of psychiatric disorders in adulthood (Fisher et al., 2013). Furthermore, PEs reported in mid compared to early adolescence (Bartels‐Velthuis, van de Willige, Jenner, van Os, & Wiersma, 2011; Kelleher et al., 2012), and those which persist over time (Dominguez et al., 2011; Wigman, Winkel, Raaijmakers et al., 2011) are associated with relatively increased odds for psychiatric and dysfunctional behavioural outcomes. Compared to PEs, there are fewer studies on NS in the general population, and there are no meta‐analyses or reviews. Like PEs, however, NS appear to be common in adolescence in the general population (Barragan, Laurens, Navarro, & Obiols, 2011; Ronald et al., 2014). As such, research on the aetiological factors that influence the presentation and the persistence of PEs and NS (PENS) in mid adolescence is informative about typical adolescent development, but may also shed light on the pathways that lead to mental illness.

While there are no published findings regarding differing trajectories of NS, several studies have identified distinct trajectories of PEs through growth mixture modelling in 10‐ to 11‐year olds (Wigman, Winkel, Raaijmakers et al., 2011) and 14‐year olds (Mackie et al., 2011), and through latent class analysis in adults (Wigman, Winkel, Jacobs et al., 2011). A meta‐analysis of cross‐age studies reported that PEs were persistent for ~20% of individuals (Linscott & van Os, 2013). As well as being associated with clinical and poor behavioural outcomes, persistence is associated with a range of risk factors including cannabis use, trauma, stressful life events and urban environment in adolescence (Cougnard et al., 2007; Wigman, Winkel, Raaijmakers et al., 2011).

Despite these findings, no studies to date have investigated the aetiological influences on the stability of PENS in mid‐to‐late adolescence. A study of adult twins hinted at a substantial genetic contribution to PE‐persistence (NS were not reported in terms of persistence). Wigman, Winkel, Jacobs et al. (2011) reported that for monozygotic (MZ) twins who experienced persistence, 49% of their co‐twins also experienced persistence, compared to 14% for dizygotic (DZ) twins, although twin model‐fitting was not conducted. A different but related conceptualization of PENS, schizotypy is viewed as being an expression of psychotic‐like behaviour at a personality level (see Linscott & van Os, 2013). One twin study has reported on the moderate stability (*r *=* *.58) of a ‘schizotypy factor’ over early to mid adolescence from ages 11–16 in 100 pairs assessed across time (Ericson, Tuvblad, Raine, Young‐Wolff, & Baker, 2011). Genetic and nonshared environmental influences explained 81% and 19% of this factor, respectively. At the second time point, variance was explained by both stable and new genetic influences (36% and 42% respectively), and stable and new nonshared environmental influences (3% and 19% respectively). While these results demonstrate that the aetiological effects influencing psychosis‐related phenotypes are both stable and dynamic, the cross‐time sample size was small for a twin study.

The largest twin study to date on adolescent PENS at a single time (using the same sample as the current study) reported heritability estimates of 15%–50% for PEs and 47%–59% for NS (Zavos et al., 2014). Common environmental influences were evident for hallucinations and parent‐rated negative symptoms (PRNS) (17%–24%), and the remainder of variance in PENS was accounted for by nonshared environment (49%–64%), and to a lesser degree for PRNS (17%). Using genotype data from unrelated individuals, SNP‐heritability has been estimated as 3%–9% in a recent genome‐wide association study (GWAS) meta‐analysis of adolescent PENS, providing further evidence of genetic effects influencing PENS (Pain et al., 2018; see also Sieradzka et al., 2015).

The current study builds on existing research by utilizing a large, representative sample of male and female twins. It encompasses four specific domains assessing PEs (paranoia, hallucinations, cognitive disorganization and grandiosity), and two assessing NS (self‐reported anhedonia, parent‐reported NS) measured over approximately 9 months in mid‐to‐late adolescence. The first aim was to estimate the extent to which genetic and environmental influences contribute to the stability of adolescent PENS. It was predicted that genetic effects would explain a substantial amount of the cross‐time covariance, and that there would be substantial overlap of genetic effects across time. It was also expected that the aetiological cross‐time correlations would be less than 1, highlighting the role of time‐specific influences. The second aim was to characterize the sample in terms of phenotypic persistence by grouping individuals according to whether their PENS persist, increase, decrease or remain low. It was predicted that persistence would be associated with higher levels of psychopathology compared to low‐scoring, increasing and decreasing scores.

## Methods

### Participants

Participants were part of the Longitudinal Experiences and Perceptions (LEAP) study, which measured PENS at age 16. LEAP is part of the Twins Early Development Study (TEDS), which has collected data from twins born during 1994 to 1996 in England and Wales across their childhood (Haworth, Davis, & Plomin, 2013). In sum, 10,868 families were invited to LEAP, of which 5,059 twin pairs and 5,076 parents returned data. A subsample of responding families was invited to LEAP phase 2 approximately 9 months later. Of 1,773 families invited for phase 2, 1,464 returned data. Demographics of the two samples are shown in Table S1. In the current study, 1,448 twin pairs have data at both time points (time 1 *M *=* *16.32 (0.68), 54.5% female, 36% MZ; time 2 *M *=* *17.06 (0.88), 58.1% female, 35% MZ). Parents and twins gave their informed consent to take part in these studies. TEDS was granted ethical approval from the Institute of Psychiatry Ethics Committee, Kings College London. See Appendix S1 for further details.

### Measures

*Psychotic experiences and negative symptoms* were measured using the Specific Psychotic Experiences Questionnaire (SPEQ; Ronald et al., 2014). The SPEQ is a validated self‐report and parent‐report assessment tool, comprising six subscales measuring mild‐to‐more severe experiences of paranoia (15 items), hallucinations (9 items), cognitive disorganization (11 items), grandiosity (8 items), hedonia (10 items, reversed to give a measure of anhedonia) and parent‐rated negative symptoms (PRNS) (10 items). See Ronald et al. (2014), and Appendix S2 for further details. *Distress* was measured using a single item following each subscale (Overall, how distressed are you by these experiences?), with exception of the anhedonia and PRNS subscales. *Depression traits* were measured using the 13‐item self‐report Short Mood and Feelings Questionnaire (SMFQ; Angold, Costello, Messer, & Pickles, 1995). *Emotional problems* and other psychopathology scales (conduct problems, hyperactivity and peer problems) were measured using the 5‐item self‐report Strengths and Difficulties Questionnaire subscales (SDQ; Goodman, 1997).

### Design

The twin design aims to disentangle the roles of genetic and environmental influences on variation in a phenotype, and on covariation between phenotypes (Boomsma, Busjahn, & Peltonen, 2002). Initial inferences can be made by comparing within‐pair MZ and DZ correlations. If MZ correlations (*r*MZ) are greater than DZ correlations (*r*DZ), additive genetic factors (A) are suggested. If *r*MZ are more than twice *r*DZ, nonadditive genetic factors (D) are implicated. Where *r*DZ are greater than half *r*MZ, shared environmental factors (C) are suggested. The extent to which *r*MZ are <1 implicates nonshared environmental influences, including measurement error (E). These correlations form the basis for quantifying the relative genetic and environmental contributions using twin model‐fitting.

### Analyses

SPSS software was used for all phenotypic analyses, using data from one randomly selected twin (per pair) with data at both time points. Untransformed data were used for descriptive statistics and frequency‐based analyses. For each SPEQ subscale, individuals were grouped as follows: ‘Low‐scoring’, time 1 and time 2 scores in the bottom 90% of scores; ‘Decreasing’, time 1 score in the top 10% and time 2 score in the bottom 90%; ‘Increasing’, time 1 score in the bottom 90% and time 2 score in the top 10%; ‘Persistent’, time 1 and time 2 scores in the top 10%. Across PENS, the top 10% of the score distribution was on average 1.41 *SD* from the mean.

Cohen's *d* was used to compare group differences in PENS, SMFQ and SDQ scores. *d* is a measure of the standardized difference between means (calculated for unequal sample sizes using; https://www.psychometrica.de/effect_size.html). Fisher's exact test was used to determine distress frequency associations between groups due to small numbers of observations in some cells. Cramer's V was used to measure the strength of the association of the chi‐squared value.

Skewed measures (paranoia, hallucinations, grandiosity and PRNS) were log‐transformed so that all skew statistics were between −1 and 1. All measures were regressed on age and sex, and residuals were standardized. A constrained saturated model, in which the means, variances and phenotypic correlation were constrained to be equal across twin order and zygosity was run using OpenMx (Boker et al., 2011) within R software (version 3.3) to derive phenotypic and twin intraclass correlations, and to test for mean and variance differences in the data.

Prior to performing bivariate twin analysis, the main assumptions of the twin model were tested using a series of saturated models, as outlined in Appendix S3. Bivariate Cholesky decompositions were fitted using OpenMx to investigate the aetiology of PENS across time points. Bivariate analysis compares MZ and DZ cross‐twin cross‐time correlations. Figure S1 shows a bivariate Cholesky decomposition solution and a correlated factors model. These are mathematically equivalent solutions and both provide useful statistics for interpretation (Loehlin, 1996). Bivariate parameter estimates derived from the Cholesky solution reflect the contribution of ACE factors to covariance (represented by the diagonal lines in the left‐hand figure in Figure S1), and aetiological correlation coefficients derived from the correlated factors model (represented by double headed arrows in the right‐hand figure in Figure S1) describe the overlap of A (*r*A), C (*r*C), and E (*r*E) influences. Opposite sex DZ twins were included in the models. The Cholesky decomposition quantifies the ACE effects at time 2 that also influence the time 1 measure, and those unique to time 2. OpenMx accounts for missing data through the use of maximum likelihood, therefore individuals with data only at time 1 were also included (*N *=* *4,870 and *N *=* *1,464 pairs at times 1 and 2 respectively).

ACE and ADE models with quantitative and qualitative sex differences were first fitted and compared to a saturated model. Only ACE models were run for hallucinations and PRNS because the twin correlations did not suggest any D influences on these scales. The −2LL (−2 times log‐likelihood) value was used to assess which of the full sex differences models fit the data best, with lower values indicating a better fit. Whichever model fit best was used to determine subsequent testing of the following models: (a) ACE or ADE with quantitative sex differences only, (b) ACE or ADE without sex differences on the aetiological correlations and (c) ACE or ADE without sex differences. Three indices of fit were generated: −2LL, Akaike's Information Criterion (AIC) and Bayesian Index Criterion (BIC). Goodness of fit for these nested models and subsequent submodels was assessed using BIC because it has been shown to outperform alternative indices for multivariate models in larger samples. Lower BIC values indicated a better fit. A BIC difference of at least 10 between two models indicates that the model with the lower BIC value is a better fit than the model to which it is being compared (Raftery, 1995).

## Results

### General descriptives

Descriptive statistics are presented in Tables S2 and S3. Table S4 shows frequencies of distress associated with PEs. Of those with some PEs, 11.8%–37.4% of individuals reported some level of distress. Between 2.1% and 10.8% reported being quite or very distressed.

### Univariate twin model‐fitting

Table 1 shows the univariate twin correlations and Tables S5–S10 show the results of testing for mean and variance differences in the data. The univariate twin estimates are reported from the bivariate twin models. Across all PENS except anhedonia, bivariate ACE models without sex differences fit the data best. An AE model without sex differences fit the data best for anhedonia (Tables S11–S16). Table 2 shows the univariate parameter estimates from these models. At each time point, genetic influences contributed moderately to the variance in PEs (heritability 22%–38%), and more so to variance in NS (heritability 45%–47%). Shared environment contributed modestly to variance in PEs (6%–19%), and to a greater extent to variance in PRNS (36%–38%). Nonshared environment contributed moderately to the variance in PENS (51%–59%), but less so for PRNS (17%–18%).

**Table 1:** Phenotypic and twin correlations

	Paranoia	Hallucinations	Cognitive disorganization	Grandiosity	Anhedonia	PRNS
Phenotypic						
Whole sample	0.63 [0.62, 0.65]	0.61 [0.59, 0.63]	0.69 [0.68, 0.71]	0.59 [0.58, 0.61]	0.63 [0.61, 0.64]	0.65 [0.63, 0.66]
Female	0.63 [0.61, 0.65]	0.61 [0.58, 0.63]	0.69 [0.67, 0.71]	0.58 [0.56, 0.60]	0.63 [0.61, 0.65]	0.65 [0.62, 0.67]
Male	0.64 [0.61, 0.67]	0.61 [0.58, 0.64]	0.69 [0.67, 0.72]	0.63 [0.60, 0.66]	0.62 [0.59, 0.65]	0.64 [0.61, 0.67]
Cross‐twin time 1						
MZM	0.45 [0.40, 0.50]	0.36 [0.30, 0.42]	0.43 [0.37, 0.49]	0.47 [0.42, 0.52]	0.47 [0.41, 0.52]	0.83 [0.81, 0.85]
MZF	0.53 [0.49, 0.57]	0.47 [0.43, 0.52]	0.46 [0.42, 0.51]	0.46 [0.41, 0.50]	0.49 [0.45, 0.54]	0.83 [0.81, 0.84]
DZM	0.26 [0.19, 0.33]	0.28 [0.20, 0.34]	0.29 [0.22, 0.35]	0.25 [0.17, 0.32]	0.21 [0.14, 0.28]	0.50 [0.45, 0.55]
DZF	0.28 [0.24, 0.31]	0.26 [0.23, 0.30]	0.21 [0.17, 0.25]	0.26 [0.22, 0.30]	0.22 [0.18, 0.25]	0.53 [0.50, 0.56]
DZOS	0.26 [0.21, 0.31]	0.23 [0.18, 0.28]	0.23 [0.18, 0.27]	0.23 [0.19, 0.28]	0.18 [0.14, 0.23]	0.50 [0.46, 0.53]
Cross‐twin time 2						
MZM	0.37 [0.26, 0.47]	0.42 [0.31, 0.51]	0.46 [0.36, 0.55]	0.37 [0.26, 0.46]	0.50 [0.40, 0.58]	0.84 [0.79, 0.87]
MZF	0.54 [0.47, 0.60]	0.59 [0.52, 0.65]	0.50 [0.43, 0.56]	0.51 [0.44, 0.58]	0.48 [0.40, 0.55]	0.84 [0.81, 0.86]
DZM	0.15 [0.02, 0.28]	0.32 [0.20, 0.43]	0.24 [0.12, 0.35]	0.27 [0.13, 0.39]	0.10 [−0.02, 0.22]	0.49 [0.39, 0.58]
DZF	0.26 [0.20, 0.32]	0.27 [0.21, 0.33]	0.15 [0.09, 0.21]	0.28 [0.22, 0.34]	0.19 [0.13, 0.25]	0.55 [0.51, 0.59]
DZOS	0.19 [0.11, 0.27]	0.21 [0.13, 0.29]	0.13 [0.05, 0.20]	0.23 [0.15, 0.31]	0.15 [0.07, 0.23]	0.50 [0.45, 0.56]
Cross‐twin cross‐time						
MZM	0.33 [0.26, 0.40]	0.34 [0.27, 0.41]	0.43 [0.36, 0.49]	0.40 [0.32, 0.46]	0.44 [0.37, 0.50]	0.57 [0.54, 0.60]
MZF	0.46 [0.41, 0.51]	0.44 [0.39, 0.49]	0.46 [0.41, 0.50]	0.45 [0.40, 0.50]	0.38 [0.33, 0.43]	0.57 [0.54, 0.59]
DZM	0.19 [0.11, 0.27]	0.24 [0.16, 0.32]	0.28 [0.20, 0.35]	0.20 [0.10, 0.28]	0.13 [0.05, 0.20]	0.29 [0.23, 0.36]
DZF	0.22 [0.18, 0.27]	0.23 [0.19, 0.27]	0.17 [0.12, 0.21]	0.22 [0.18, 0.26]	0.18 [0.14, 0.22]	0.36 [0.32, 0.39]
DZOS	0.18 [0.13, 0.24]	0.19 [0.14, 0.24]	0.17 [0.11, 0.22]	0.20 [0.14, 0.25]	0.17 [0.11, 0.22]	0.32 [0.28, 0.36]

A full constrained saturated model was used to obtain phenotypic intraclass correlations for males and females. A reduced model was fit to obtain intraclass correlations collapsed by sex. Twin intraclass correlations were obtained from the full constrained saturated model.

DZF, Dizygotic females; DZM, Dizygotic males; DZOS, Dizygotic opposite sex; MZF, Monozygotic females; MZM, Monozygotic males; PRNS, Parent‐rated negative symptoms.

**Table 2:** Parameter estimates for best‐fitting bivariate Cholesky solutions

	Standardized univariate estimates time 1			Standardized univariate estimates time 2			Bivariate heritability, bivariate shared environment, bivariate nonshared environment			Proportion of phenotypic correlation explained by A, C and E		
	A	C	E	A	C	E	A	C	E	A	C	E
Paranoia	0.28 [0.22, 0.34]	0.19 [0.15, 0.23]	0.53 [0.50, 0.56]	0.32 [0.22, 0.43]	0.12 [0.05, 0.19]	0.55 [0.50, 0.60]	0.25 [0.19, 0.32]	0.12 [0.07, 0.17]	0.23 [0.20, 0.27]	0.42 [0.31, 0.53]	0.20 [0.12, 0.27]	0.38 [0.32, 0.44]
Hallucinations	0.22 [0.16, 0.28]	0.19 [0.15, 0.23]	0.59 [0.56, 0.63]	0.33 [0.23, 0.43]	0.16 [0.09, 0.22]	0.51 [0.46, 0.57]	0.22 [0.16, 0.29]	0.14 [0.10, 0.19]	0.22 [0.18, 0.25]	0.38 [0.27, 0.49]	0.25 [0.17, 0.32]	0.37 [0.31, 0.43]
Cognitive disorganization	0.27 [0.21, 0.33]	0.15 [0.11, 0.19]	0.58 [0.55, 0.62]	0.38 [0.30, 0.45]	0.06 [0.02, 0.11]	0.56 [0.51, 0.62]	0.3 [0.24, 0.35]	0.09 [0.05, 0.12]	0.26 [0.23, 0.30]	0.46 [0.37, 0.54]	0.13 [0.08, 0.19]	0.41 [0.36, 0.46]
Grandiosity	0.26 [0.20, 0.32]	0.18 [0.13, 0.22]	0.57 [0.53, 0.60]	0.26 [0.16, 0.36]	0.19 [0.12, 0.25]	0.56 [0.50, 0.62]	0.25 [0.18, 0.31]	0.13 [0.09, 0.18]	0.19 [0.16, 0.23]	0.43 [0.32, 0.54]	0.23 [0.16, 0.30]	0.34 [0.28, 0.40]
Anhedonia	0.47 [0.44, 0.50]	‐	0.53 [0.50, 0.56]	0.46 [0.40, 0.51]	–	0.54 [0.49, 0.60]	0.37 [0.33, 0.41]	–	0.22 [0.19, 0.26]	0.62 [0.57, 0.68]	–	0.38 [0.32, 0.43]
PRNS	0.46 [0.42, 0.50]	0.36 [0.33, 0.39]	0.18 [0.17, 0.20]	0.45 [0.39, 0.51]	0.38 [0.33, 0.43]	0.17 [0.15, 0.20]	0.34 [0.30, 0.38]	0.21 [0.17, 0.25]	0.08 [0.06, 0.09]	0.54 [0.48, 0.60]	0.33 [0.28, 0.39]	0.12 [0.10, 0.15]

A, Additive genetic effects; C, Common environmental effects; E, Nonshared environmental effects; PRNS, Parent‐rated negative symptoms; 95% CI in parentheses.

### Bivariate twin model‐fitting

Table 1 shows the phenotypic cross‐time correlations (*r *=* *.59–.69) and cross‐twin cross‐time correlations. Cross‐twin cross‐time *r*MZ were higher than *r*DZ for all PENS suggesting genetic influences, and cross‐twin cross‐time *r*MZ were all less than 1, suggesting nonshared environment on the cross‐time covariation. For hallucinations and PRNS, cross‐twin cross‐time *r*DZ were greater than half *r*MZ, suggesting shared environment on the cross‐time covariance. *r*DZ were less than half *r*MZ for female paranoia, cognitive disorganization and grandiosity, and across sexes for anhedonia, suggesting some nonadditive genetic effects.

Table S17 shows the Cholesky estimates. Across PENS, between 42% and 58% of the variance at time 2 was accounted for by aetiological influences carried over from time 1. Specifically, 25%–38% of the variance in each measure at time 2 was accounted for by genetic influences carried across time, 0%–13% was due to shared environment, and 3%–14% was due to nonshared environment. Aetiological influences unique to time 2 were highest for nonshared environment across PENS (42%–49%, except PRNS, 14%).

Genetic correlations indicated substantial overlap in genetic influences across time (*r*A* *=* *.77–1.00) (Table S18). The high *r*C estimates across PENS suggest considerable overlap in C influences (*r*C* *=* *.59–1.00). Moderate *r*E suggest that E influences across time partially overlap (*r*E* *=* *.36–.49). Table** 2 shows the bivariate parameter estimates. The proportion of the phenotypic correlation that was explained by genetic influences was 0.38–0.46 for PEs and 0.54–0.62 for NS. The proportion of the phenotypic correlation that was explained by shared environmental influences was 0.13–0.33, except for anhedonia which showed no C. The proportion of the phenotypic correlation that was explained by nonshared environmental influences was 0.34–0.41, although less for PRNS (0.12).

### Cross‐time phenotypic subgroup analysis

Table S19 shows the descriptives of the phenotypic persistence subgroups. For individuals with high time 1 PENS, means were significantly higher for persistent compared to decreasing groups (*d *=* *0.31–0.56, except grandiosity). For individuals with low time 1 PENS, means were significantly higher for the increasing compared to low‐scoring groups (*d *=* *1.08–1.61). Between 5.5% and 8.1% of individuals had persistently high PENS (Table S19).

Table 3 shows that for all PEs except grandiosity, point estimates suggested that the persistent group reported being quite or very distressed more often than the other groups, and reported being not distressed to a lesser extent. Fisher's exact test and Cramer's *V* statistics (0.15–0.46) were significant (*p *≤ .001) for comparisons between the low‐scoring and increasing groups, the persistent and low‐scoring groups, and between the persistent and increasing groups, but not between the persistent and decreasing groups.

**Table 3:** Frequency differences for distress at time 1 by group

	N with PE score > 0 and distress data	PE mean score at time 1 (*SD*)	Not distressed	A bit distressed	Quite/very distressed	Comparison	Fisher's exact test (*p*)	Cramer's V (*p*)
Paranoia								
Low‐scoring (LS)	854	11.61 (8.65)	620 (72.6%)	196 (23.0%)	38 (4.4%)	LS versus P	125.56 (<.001)**	0.45 (<.001)**
Increasing (I)	46	23.25 (8.63)	17 (37.0%)	22 (47.8%)	7 (15.2%)	LS versus I	25.97 (<.001)**	0.18 (<.001)**
Decreasing (D)	53	42.91 (6.02)	14 (26.4%)	24 (45.3%)	15 (28.3%)	D versus P	5.34 (.07)	0.21 (.07)
Persistent (P)	66	45.73 (8.28)	9 (13.6%)	26 (39.4%)	31 (47.0%)	I versus P	15.15 (.001)**	0.36 (.001)**
Hallucinations								
Low‐scoring (LS)	572	5.25 (4.23)	484 (84.6%)	78 (13.6%)	10 (1.7%)	LS versus P	83.76 (<.001)**	0.43 (<.001)**
Increasing (I)	63	11.31 (4.54)	41 (65.1%)	21 (33.3%)	1 (1.6%)	LS versus I	14.34 (.001)**	0.16 (.001)**
Decreasing (D)	71	22.65 (5.42)	36 (50.7%)	25 (35.2%)	10 (14.1%)	D versus P	4.26 (.11)	0.18 (.11)
Persistent (P)	65	24.20 (5.97)	22 (33.8%)	28 (43.1%)	15 (23.1%)	I versus P	20.10 (<.001)**	0.39 (<.001)**
Cognitive disorganization								
Low‐scoring (LS)	765	4.00 (2.11)	556 (72.7%)	172 (22.5%)	37 (4.8%)	LS versus P	142.58 (<.001)**	0.46 (<.001)**
Increasing (I)	59	6.53 (1.61)	29 (49.2%)	21 (35.6%)	9 (15.3%)	LS versus I	16.66 (<.001)**	0.15 (.001)**
Decreasing (D)	77	9.61 (0.69)	23 (29.9%)	33 (42.9%)	21 (27.3%)	D versus P	5.66 (.06)	0.18 (.06)
Persistent (P)	96	10.09 (0.76)	17 (17.7%)	38 (39.6%)	41 (42.7%)	I versus P	20.92 (<.001)**	0.37 (<.001)**
Grandiosity								
Low‐scoring (LS)	810	4.54 (2.99)	699 (86.3%)	89 (11.0%)	22 (2.7%)	LS versus P	1.29 (.54)	0.03 (.67)
Increasing (I)	40	7.72 (2.93)	28 (70.0%)	10 (25.0%)	2 (5.0%)	LS versus I	7.89 (.02)*	0.10 (.02)*
Decreasing (D)	58	16.66 (2.92)	51 (87.9%)	5 (8.6%)	2 (3.4%)	D versus P	0.60 (.79)	0.07 (.79)
Persistent (P)	66	17.16 (2.71)	55 (83.3%)	8 (12.1%)	3 (4.5%)	I versus P	3.08 (.22)	0.17 (.28)

*N*, Number of individuals; One randomly selected twin per pair included in analyses; Data shown for sample included in phenotypic analyses who provided data at both time points; Fisher's exact test of independence; Cramer's V measure of effect size (square root of the *x2* statistic divided by the sample size multiplied by the lesser number of categories in either variable minus 1); Monte Carlo *p* values based on 10,000 sampled tables.

D, Decreasing group; I, Increasing group; LS, Low‐scoring group; P, Persistent group; PE, Psychotic experiences.

**p < .001;*p < .05

Across PENS, the persistent group showed a pattern of having higher point estimates for both depression traits and emotional problems than the increasing, decreasing and low‐scoring groups. The persistent group had more depression traits (*d *=* *0.47–1.67, except grandiosity), and emotional problems (*d *=* *0.47–1.47, except grandiosity and anhedonia) compared to low‐scorers as indicated by significantly larger effect sizes, and for some subscales such as paranoia, differences reached significance between the persistent and the increasing/decreasing groups (Tables S20 and S21).

In some additional analyses, the persistence groups were compared for the other psychopathology subscales of the SDQ, namely hyperactivity, conduct problems and peer problems. As shown in Tables S22–S24, the same pattern was shown as for depression traits and emotional problems, with the persistent group having more conduct problems (*d *=* *0.28–0.95), hyperactivity (*d *=* *0.14–1.50), and peer problems (*d *=* *0.48–1.22, except grandiosity), compared to low‐scorers (indicated by significantly larger effect sizes), and for some subscales such as paranoia and cognitive disorganization, significant differences were apparent between the persistent and the increasing/decreasing groups. Correlations between PENS, SMFQ and SDQ scales are shown in Table S25.

## Discussion

This is the first study to investigate the genetic and environmental influences on the stability of PENS in a large sample in mid‐to‐late adolescence. Over a period of ~9 months at ages 16–17 years, PENS showed considerable phenotypic stability as reflected in the high phenotypic correlations. This stability was influenced by both genetic and environmental factors, with genetic and nonshared environmental influences explaining a similar proportion of the relationship between PEs across time, and genetic influences explaining a larger proportion of the stability of NS. Of the genetic and common environmental influences that contributed to stability, most were shared across time, although overlap of nonshared environmental effects was much lower. Nonshared environmental influences at the later time point contributed to the imperfect stability of PENS.

Individuals with persistent PENS reported higher levels of PENS (with exception of grandiosity) than individuals with PENS that were either increasing, decreasing or consistently low. The persistent group also tended to have more distress associated with their PEs, and higher levels of depression traits and emotional problems and other psychopathology at baseline compared to the other groups. The majority of comparisons showed a significant effect size when comparing the persistent group with the increasing, decreasing or low‐scoring groups. The direction of effect was such that the persistent group was the more impaired or distressed group. It is noted that not all comparisons had a significant effect size, and in particular, being in the group characterized by persistence of grandiosity was not associated with more distress and psychopathology. This is broadly in line with past findings suggesting that grandiosity does not always link with other psychopathology at this age (Ronald et al., 2014). The grandiosity scale appeared similar to the other PENS scales in terms of its high internal consistency and its positive skew. Future work should explore whether this distinct pattern shown by grandiosity is specific to adolescence.

Our finding that genetic influences contribute moderately to the stability of PENS in adolescence is broadly in line with findings by Ericson et al. (2011), who reported a strong genetic component contributing to the stability of schizotypy (a related phenotype), albeit on a smaller and younger sample. Unlike the Ericson et al. study, we also identified modest shared environmental influences and moderate nonshared environmental influences for all PEs. Our study also extended this work by reporting on stability across specific psychotic experiences and negative symptoms.

The results highlight the role of environmental factors in influencing how adolescent PENS develop, which adds to existing research that has shown the importance of environmental factors at single time points (Hur, Cherny, & Sham, 2012; Zavos et al., 2014; Zhou et al., 2018). Of particular interest in this context are our results that nonshared environment contributes to more than a third of the stability of PENS (except PRNS). These findings concur broadly with findings that specific environmental risks such as trauma, cannabis use and stressful life events are associated with persistent PEs in adolescence (Cougnard et al., 2007; Wigman, Winkel, Raaijmakers et al., 2011). Furthermore, our results suggest that some nonshared environmental influences are time‐specific. Whilst estimates for nonshared environment also include measurement error, the results suggest that in part, PENS are influenced by time‐specific factors not shared between family members. This is in line with findings suggesting that some nonshared environmental effects at least prior to adulthood are transitory, in contrast to shared environmental and genetic effects which are more stable over time (Burt, Klahr, & Klump, 2015).

The modest contribution of shared environment to stability of most of the PENS studied (notably not anhedonia) can be considered in the light of epidemiological findings that have identified urbanicity as a risk factor for persistence in individuals in the general population reporting PEs at baseline (Cougnard et al., 2007). Whilst the findings cannot be used to draw conclusions about the exact nature of common environmental influences, they are more generally reflective of findings that shared environments explain less variance in behavioural phenotypes than nonshared environments (Plomin, 2011). The higher proportion of phenotypic stability explained by shared environment for PRNS may be influenced by the effect of having the same rater across twins.

Psychological difficulties such as distress, depression traits and emotional problems and other psychopathology were elevated at baseline in those who followed a persistent path in terms of PENS. This suggests that individuals who go on to experience high levels of PENS over time are more likely to be suffering with current psychological disturbance as well as being at increased risk of later psychopathology (Dominguez et al., 2011; Wigman, Winkel, Raaijmakers et al., 2011).

### Strengths and limitations

It is a key strength of this study that data from over 4,800 twin pairs was used, building on existing research that has relied on smaller samples. Further, the study utilized a validated measurement tool encompassing measurement of four individual dimensions of PEs and two of NS. In the light of this, it is a limitation that the time 2 sample was smaller than the time 1 sample, and that not more time points were available. However, our results broadly concur with other findings that modelled data on younger and older samples assessed across three time points (Wigman, Winkel, Jacobs et al., 2011; Wigman, Winkel, Raaijmakers et al., 2011). Future work should seek to employ both researcher‐ and data‐driven methods in order to cross‐validate the results.

## Conclusion

Both genetic and environmental influences contribute to the considerable stability of adolescent PENS in mid‐to‐late adolescence. There are also some dynamic influences particularly via nonshared environments. Individuals who will go on to report persistent PENS are more likely to experience other psychological difficulties such as distress, depression traits and other psychopathology. In conjunction with epidemiological findings in the field, the findings presented here speak of the importance of measuring adolescent PENS over time.

**Key points**

- Persistence of psychotic experiences and negative symptoms (PENS) is known to reflect heightened risk for psychiatric disorders, but the causes of this persistence are unknown.
- PENS were found to be largely stable over a period of 9 months in adolescence.
- Persistent PENS tended to be associated with greater levels of distress and other psychopathology at baseline compared to groups with transitory or low levels of PENS.
- Genetic and environmental influences contributed to the stability of PENS in adolescence.
- Time‐specific effects acted primarily via nonshared environment. The imperfect stability of PENS was at least partly due to new nonshared environmental influences occurring over time.

## Supporting information


**Appendix S1.** Study details.
**Appendix S2.** The Specific Psychotic Experiences Questionnaire (SPEQ; Ronald et al., 2014).
**Appendix S3.** Assumptions testing.
**Figure S1.** Bivariate Cholesky decomposition solution (left‐hand figure) and correlated factors model (right‐hand figure) path diagrams.
**Table S1.** Frequency and mean differences in demographics of main sample and follow‐up sample.
**Table S2.** Descriptives for psychotic experiences and negative symptoms subscales.
**Table S3.** Descriptives for psychotic experiences and negative symptoms subscales split by sex and zygosity.
**Table S4.** Frequencies of distress associated with psychotic experiences.
**Table S5–S10.** Results of testing for mean and variance differences in the data.
**Table S11–S16.** Bivariate twin model statistics.
**Table S17.** Cholesky estimates.
**Table S18.** Genetic and environmental correlations for best‐fitting bivariate models.
**Table S19.** Descriptives and mean differences for psychotic experiences and negative symptoms subscales at time 1 by group.
**Table S20–S21.** Mean differences for depression traits and emotional problems at time 1 by group.
**Table S22–S24.** Mean differences for conduct problems, hyperactivity and peer problems at time 1 by group.
**Table S25.** Correlations for psychotic experiences and negative symptoms with depression traits and SDQ emotional problems and other SDQ subscales.
**Table S26.** Descriptive statistics for depression traits and psychopathology subscales.
