# Supplementary material for: Genetic and environmental influences on the stability of psychotic experiences and negative symptoms in adolescence
Source: J Child Psychol Psychiatry. 2019 Apr 7;60(7):784–92. doi: 10.1111/jcpp.13045 (PMC6619355; doi:10.1111/jcpp.13045)
Supplement: Supplementary file 1 — Appendix S1. Study details. Appendix S2. The Specific Psychotic Experiences Questionnaire (SPEQ; Ronald et al., 2014). Appendix S3. Assumptions testing. Figure S1. Bivariate Cholesky decomposition solution (left‐hand figure) and correlated factors model (right‐hand figure) path diagrams. Table S1. Frequency and mean differences in demographics of main sample and follow‐up sample. Table S2. Descriptives for psychotic experiences and negative symptoms subscales. Table S3. Descriptives for psychotic experiences and negative symptoms subscales split by sex and zygosity. Table S4. Frequencies of distress associated with psychotic experiences. Table S5–S10. Results of testing for mean and variance differences in the data. Table S11–S16. Bivariate twin model statistics. Table S17. Cholesky estimates. Table S18. Genetic and environmental correlations for best‐fitting bivariate models. Table S19. Descriptives and mean differences for psychotic experiences and negative symptoms subscales at time 1 by group. Table S20–S21. Mean differences for depression traits and emotional problems at time 1 by group. Table S22–S24. Mean differences for conduct problems, hyperactivity and peer problems at time 1 by group. Table S25. Correlations for psychotic experiences and negative symptoms with depression traits and SDQ emotional problems and other SDQ subscales. Table S26. Descriptive statistics for depression traits and psychopathology subscales. [file JCPP-60-784-s001.pdf]

This is supplementary information for 'Genetic and environmental influences on the stability of psychotic experiences and negative symptoms in adolescence' by Laura Havers et al.

Appendix S1. *Study details*

Initially TEDS contacted 16,810 families when the twins were 18 months old, of which 13,694 families responded. In sum, 10,868 families were invited to participate in LEAP. Families were not invited either because of study withdrawal, non-return of data, contact difficulties, or medical exclusion. From this, 5,059 twin pairs and 5,076 parents returned data. For LEAP phase 2, 1,773 families were invited and 1,464 families returned data. In the current study, 5,163 twin pairs had LEAP data available, of which 293 pairs were excluded due to non-consent, unknown zygosity, perinatal complications, severe medical disorder, or an autism diagnosis. For the phenotypic stability analyses, participants with data at both ages were included (N = 1,448). For the twin model-fitting analyses, N = 4,870 twin pairs at time 1 and N = 1,464 pairs at time 2 were included in the analyses.

Families were rewarded for their participation in these stages of data collection. Consent from parents and twins was required separately at age 16.

Appendix S2. *The Specific Psychotic Experiences Questionnaire (SPEQ; Ronald et al., 2014)*

SPEQ subscales were developed from adult scales, through rewording and selection of items. Age-appropriateness and content validity was established through expert clinical consultation and piloting (Ronald et al., 2014). Subscales were derived from Principal Components Analysis. Validity was assessed using family history data and testing agreement with another measure of adolescent PEs (Psychosis-Like Symptoms scale; Zammit, Owen, Evans, Heron, & Lewis, 2011). Differences in the mean scores of singletons and the averaged mean across twin pairs were not significant across PENS except for paranoia ($p < .05$), which was not significant after correcting for multiple testing ($p < .01$) (see Ronald et al., 2014).

The SPEQ is comprised of six subscales: *Paranoia:* Fifteen (15) items measured the frequency of paranoid experiences, for example, "How often have you thought, people might be conspiring against me"? *Hallucinations:* Nine (9) items measured the frequency of hallucinatory experiences, for example, "How often do you hear sounds or music that people near you don't hear"? *Cognitive disorganization:* Eleven (11) items measured the presence of experiences over the past month, for example, "Are you easily confused if too much happens at the same time"? *Grandiosity:* Eight (8) items measured the frequency of grandiose experiences, for example, "I have special abilities that others do not". *Hedonia:* Ten (10) items measured anticipatory pleasure, for example, "When something exciting is coming up in my life, I really look forward to it". Scores were reversed to give a measure of anhedonia. *Parent-rated negative symptoms (PRNS):* Parents reported on 10 items reflecting negative symptoms in each twin, for example, "He/she usually gives brief, one word replies to questions, even if encouraged to say more".

Appendix S3. *Assumptions testing*

Prior to carrying out bivariate twin analysis, assumptions of the twin design were tested for each individual measure. Twin models assume that the mean value of and variance in a given trait does not differ between twins within a pair or across zygosity. To test these assumptions, we first fitted a baseline fully saturated model in which the means and variances were permitted to differ across individuals within a pair and across zygosity. Each assumption was then tested through three submodels: a) a model that equated means within twin pairs; b) a model that equated variances within twin pairs; and c) a model that equated means and variances across twin order. Significant results indicate a violation of a given assumption. For all measures, except for parent-rated negative symptoms, means and variances could be equated across twin order and zygosity. The violation of these assumptions for parent-rated negative symptoms could arise from the relatively strong skew of the scale, which can lead to unequal means across twin order. In addition, a violation of the assumption of equal variances across zygosity can arise when the manner in which parents rate one twin affects how they rate their co-twin (Neale & Maes, 2004). These effects, referred to as sibling contrast effects, are reported fairly commonly for parent-rated measures of child psychopathology (e.g., Ronald et al., 2008, 2014).

Two additional tests were conducted using submodels of the saturated model. First, it was tested whether males and females within opposite-sex pairs had equal means to males and females within same-sex pairs. It was also tested whether it was possible to equate the means and variances across sexes in same-sex pairs, to test for sex differences. These models generally suggested sex differences, except for grandiosity and anhedonia. These results are shown in tables S5-S10.

Figure S1. Bivariate Cholesky decomposition solution (left-hand figure) and correlated factors model (right-hand figure) path diagrams.

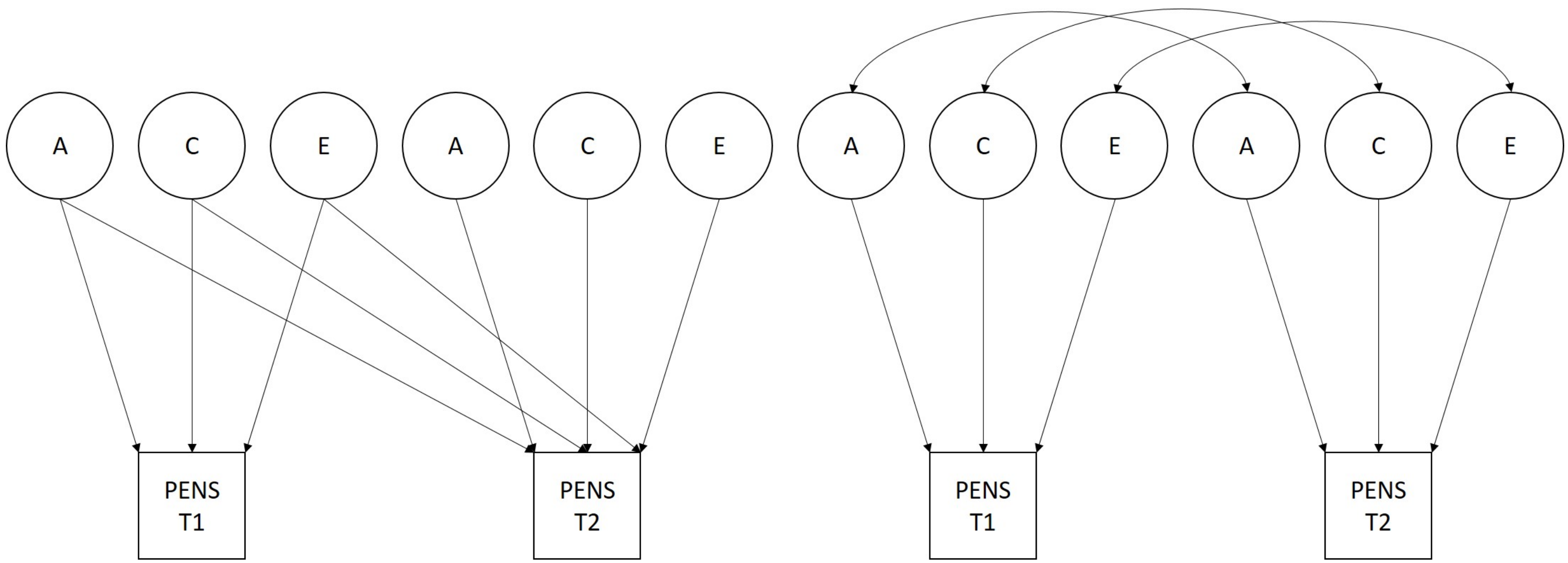

Note. A, Additive genetic influences; C, shared environmental influences; E, nonshared environmental influences; PENS, psychotic experiences and negative symptom traits; T1, time 1; T2, time 2.

Table S1. *Frequency and mean differences in demographics of main sample and follow-up sample*

	Main sample	Follow-up sample
General exclusion at 16	315 (6.9%)	39 (2.6%)
Medical exclusion at 16	170 (3.7%)	39 (2.6%)
Perinatal outlier exclusion at first contact	97 (2.1%)	0 (0.0%)
Ethnic origin at first contact (non-white)	311 (6.9%)	108 (7.3%)
Main language spoken at home at first contact (other / English plus other)	147 (3.2%)	61 (4.1%)
Standardised SES composite score at first contact (mean / *SD*)	0.19 (0.99)	0.19 (0.99)

Note. N individuals included in analysis: Main sample = 4,535, follow-up sample = 1,487; SES, Socio-economic status; General exclusion applied if no data is available from first contact, for medical exclusion purposes, for perinatal outliers, and for unknown sex or zygosity; Medical exclusion applied if any medical exclusion has been recorded up to and including the age of age of 16.

Table S2. *Descriptives for psychotic experiences and negative symptoms subscales*

	Paranoia		Hallucinations		Cognitive disorganization		Grandiosity		Anhedonia		PRNS	
	Time 1	Time 2	Time 1	Time 2	Time 1	Time 2	Time 1	Time 2	Time 1	Time 2	Time 1	Time 2
N	1,446	1,437	1,448	1,439	1,446	1,440	1,446	1,439	1,409	1,400	1,446	1,433
Missing	2	11	0	9	2	8	2	8	39	48	2	15
Scale range	0-75	0-75	0-45	0-45	0-11	0-11	0-24	0-24	0-50	0-50	0-30	0-30
Observed	0-65	0-72	0-45	0-45	0-11	0-11	0-24	0-24	0-49	0-47	0-30	0-30
Mean	15.21	14.80	6.39	6.78	4.58	4.50	5.63	4.75	16.97	16.93	3.71	3.72
95% CI	[14.51, 15.91]	[14.09, 15.52]	[5.98, 6.79]	[6.39, 7.17]	[4.42, 4.75]	[4.34, 4.66]	[5.36, 5.90]	[4.50, 5.00]	[16.50, 17.43]	[16.50, 17.35]	[3.45, 3.97]	[3.47, 3.96]
Median	11.00	11.00	3.00	4.00	4.00	4.00	4.00	3.00	15.00	16.00	2.00	2.00
Mode	0	0	0	0	4	3	1	1	14	15	0	0
SD	13.60	13.81	7.82	7.54	3.21	3.11	5.26	4.78	8.92	8.05	5.08	4.73
Variance	184.82	190.65	61.11	56.87	10.29	9.67	27.70	22.84	79.50	64.75	25.83	22.34
Skewness	1.18	1.40	1.56	1.56	.34	.31	1.30	1.40	.66	.50	1.93	2.00
Kurtosis	.78	1.88	2.15	2.54	-.96	-.95	1.15	1.83	.01	.07	3.72	4.62
Cronbach's α	.94	.95	.91	.90	.82	.81	.90	.89	.74	.72	.89	.89

Note. N, Number of individuals; PRNS, Parent-rated negative symptoms; One randomly selected twin per pair included in analyses; Data shown for sample included in phenotypic analyses who provided data at both time points.

Table S3. *Descriptives for psychotic experiences and negative symptoms subscales split by sex and zygosity*

	Paranoia			Hallucinations			Cognitive disorganization			Grandiosity			Anhedonia			PRNS		
	N	*M*	*SD*	*N*	*M*	*SD*	*N*	*M*	*SD*	*N*	*M*	*SD*	*N*	*M*	*SD*	*N*	*M*	*SD*
Time 1																		
MZM	187	13.77	13.67	187	5.42	7.71	187	3.99	3.13	187	6.00	5.17	182	19.49	9.38	187	3.66	4.69
MZF	321	15.56	13.56	322	6.80	8.15	322	4.96	3.21	321	5.01	4.95	312	15.04	8.07	322	3.42	4.87
DZM	178	15.22	13.21	178	6.48	7.52	176	4.17	3.21	178	6.45	5.78	172	20.19	9.34	177	4.39	5.71
DZF	277	13.85	12.79	277	6.23	7.92	277	4.85	3.22	277	5.06	5.06	274	15.00	8.10	277	3.37	5.07
DZOS	483	16.30	14.11	484	6.54	7.68	484	4.56	3.19	483	5.93	5.37	469	17.25	8.98	483	3.86	5.12
Time 2																		
MZM	186	12.38	12.21	186	6.42	8.07	187	3.71	2.96	187	5.90	5.36	180	18.57	8.26	185	3.90	4.64
MZF	320	15.49	14.91	321	6.80	7.51	321	5.06	3.26	321	4.12	4.46	308	15.42	7.39	319	3.52	4.45
DZM	177	14.92	13.99	177	7.27	8.04	176	4.29	3.10	176	5.99	5.27	175	19.52	8.43	177	4.37	5.20
DZF	275	14.68	13.80	275	6.70	7.15	276	4.72	3.04	275	4.13	4.36	270	14.82	7.66	274	3.15	4.58
DZOS	479	15.31	13.51	480	6.76	7.40	480	4.39	3.04	480	4.62	4.64	467	17.53	7.99	478	3.86	4.82

Note. N, Number of individuals; MZM, Monozygotic males; MZF, Monozygotic females; DZM, Dizygotic males; DZF, Dizygotic females; DZOS, Dizygotic opposite sex; PRNS, Parent-rated negative symptoms; One randomly selected twin per pair included in analyses; Data shown for sample included in phenotypic analyses who provided data at both time points.

Table S4. *Frequencies of distress associated with psychotic experiences*

	N with PE score >0 and distress data	Not distressed	A bit distressed	Quite / very distressed
Paranoia time 1	1,026	666 (64.9%)	268 (26.1%)	92 (9.0%)
Paranoia time 2	1,335	912 (68.3%)	320 (24.0%)	103 (7.7%)
Hallucinations time 1	776	586 (75.5%)	153 (19.7%)	37 (4.8%)
Hallucinations time 2	1,127	958 (85.0%)	145 (12.9%)	24 (2.1%)
Cognitive disorganization time 1	1,001	627 (62.6%)	266 (26.6%)	108 (10.8%)
Cognitive disorganization time 2	1,283	922 (71.9%)	289 (22.5%)	72 (5.6%)
Grandiosity time 1	979	837 (85.5%)	112 (11.4%)	30 (3.1%)
Grandiosity time 2	1,221	1,077 (88.2%)	105 (8.6%)	39 (3.2%)

Note. N, Number of individuals; Distress data included only where psychotic experiences total subscale scores >0 at relative time-point; One randomly selected twin per pair included in analyses; Distress data not available for negative symptoms. PE, Psychotic experiences scale.

Table S5. *Assumption testing for paranoia*

					Comparative fit with model in row above				Comparative fit with saturated model			
Model	-2LL	*df*	Parameters	BIC	$^{\Delta}\chi^2$	$^{\Delta}df$	*p*	AIC	$^{\Delta}\chi^2$	$^{\Delta}df$	*p*	AIC
Saturated	43,244.21	16,398	70	-100,473.86	-	-	-	-	-	-	-	-
a	43,261.15	16,406	62	-100,527.04	16.94	8	.03	0.94	16.94	8	.03	0.94
b	43,266.15	16,414	54	-100,592.15	5.01	8	.76	-10.99	21.94	16	.15	-10.06
c	43,282.94	16,422	46	-100,645.48	16.78	8	.03	0.78	38.73	24	.03	-9.27
d	43,323.36	16,430	38	-100,675.17	40.43	8	<.001	24.43	79.15	32	<.001	15.15
e	43,335.36	16,434	34	-100,698.23	11.99	4	.02	3.99	91.15	36	<.001	19.15

Note. Model definitions: a, equating means within twin pairs for same-sex pairs. b, equating variances within twin pairs for same-sex pairs. c, equating means and variances across zygosity for same-sex pairs (separating means and variances by sex). d, equating males in opposite-sex pairs with males in same-sex pairs (and the same for females). e, testing for sex differences in means and variances. −2LL, likelihood-ratio test (minus two times the log-likelihood of the data); BIC, Bayesian Information Criteria; AIC, Akaike's Information Criteria*; df,* degrees of freedom; $^{\Delta}\chi^2$, difference in −2LL statistic between models; $^{\Delta}df$, difference in degrees of freedom between models; AIC calculated as $^{\Delta}\chi^2$ minus twice $^{\Delta}df$.

Table S6. *Assumption testing for hallucinations*

					Comparative fit with model in row above				Comparative fit with saturated model			
Model	-2LL	*df*	Parameters	BIC	$^{\Delta}\chi^2$	$^{\Delta}df$	*p*	AIC	$^{\Delta}\chi^2$	$^{\Delta}df$	*p*	AIC
Saturated	43,756.14	16,413	70	-100,093.40	-	-	-	-	-	-	-	-
a	43,770.46	16,421	62	-100,149.19	14.32	8	.07	-1.68	14.32	8	.07	-1.68
b	43,773.82	16,429	54	-100,215.95	3.36	8	.91	-12.64	17.68	16	.34	-14.32
c	43,780.97	16,437	46	-100,278.92	7.15	8	.52	-8.85	24.83	24	.42	-23.17
d	43,821.65	16,445	38	-100,308.34	40.69	8	<.001	24.69	65.52	32	<.001	1.52
e	43,841.01	16,449	34	-100,324.04	19.36	4	<.001	11.36	84.88	36	<.001	12.88

Note. Model definitions: a, equating means within twin pairs for same-sex pairs. b, equating variances within twin pairs for same-sex pairs. c, equating means and variances across zygosity for same-sex pairs (separating means and variances by sex). d, equating males in opposite-sex pairs with males in same-sex pairs (and the same for females). e, testing for sex differences in means and variances. −2LL, likelihood-ratio test (minus two times the log-likelihood of the data); BIC, Bayesian Information Criteria; AIC, Akaike's Information Criteria*; df,* degrees of freedom; $^{\Delta}\chi^2$, difference in −2LL statistic between models; $^{\Delta}df$, difference in degrees of freedom between models; AIC calculated as $^{\Delta}\chi^2$ minus twice $^{\Delta}df$.

Table S7. *Assumption testing for cognitive disorganization*

					Comparative fit with model in row above				Comparative fit with saturated model			
Model	-2LL	*df*	Parameters	BIC	$^{\Delta}\chi^2$	$^{\Delta}df$	*p*	AIC	$^{\Delta}\chi^2$	$^{\Delta}df$	*p*	AIC
Saturated	42,957.75	16,401	70	-100,786.61	-	-	-	-	-	-	-	-
a	42,971.74	16,409	62	-100,842.73	14.00	8	.08	-2.00	14.00	8	.08	-2.00
b	42,978.96	16,417	54	-100,905.63	7.22	8	.51	-8.78	21.21	16	.17	-10.79
c	42,994.45	16,425	46	-100,960.26	15.49	8	.05	0.51	38.70	24	.05	-9.30
d	43,064.49	16,433	38	-100,960.33	70.04	8	<.001	54.04	106.74	32	<.001	42.74
e	43,092.82	16,437	34	-100,967.06	28.33	4	<.001	20.33	135.07	36	<.001	63.07

Note. Model definitions: a, equating means within twin pairs for same-sex pairs. b, equating variances within twin pairs for same-sex pairs. c, equating means and variances across zygosity for same-sex pairs (separating means and variances by sex). d, equating males in opposite-sex pairs with males in same-sex pairs (and the same for females). e, testing for sex differences in means and variances. −2LL, likelihood-ratio test (minus two times the log-likelihood of the data); BIC, Bayesian Information Criteria; AIC, Akaike's Information Criteria*; df,* degrees of freedom; $^{\Delta}\chi^2$, difference in −2LL statistic between models; $^{\Delta}df$, difference in degrees of freedom between models; AIC calculated as $^{\Delta}\chi^2$ minus twice $^{\Delta}df$.

Table S8. *Assumption testing for grandiosity*

					Comparative fit with model in row above				Comparative fit with saturated model			
Model	-2LL	*df*	Parameters	BIC	$^{\Delta}\chi^2$	$^{\Delta}df$	*p*	AIC	$^{\Delta}\chi^2$	$^{\Delta}df$	*p*	AIC
Saturated	43,969.74	16,402	70	-99,783.39	-	-	-	-	-	-	-	-
a	43,973.67	16,410	62	-99,849.57	3.93	8	.86	-12.07	3.93	8	.86	-12.07
b	43,978.71	16,418	54	-99,914.64	5.04	8	.75	-10.96	8.97	16	.92	-23.03
c	43,989.83	16,426	46	-99,973.64	11.12	8	.19	-4.88	20.09	24	.69	-27.91
d	43,998.76	16,434	38	-100,034.82	8.93	8	.34	-7.07	29.02	32	.62	-34.98
e	44,001.92	16,438	34	-100,066.73	3.16	4	.53	-4.84	32.18	36	.65	-39.82

Note. Model definitions: a, equating means within twin pairs for same-sex pairs. b, equating variances within twin pairs for same-sex pairs. c, equating means and variances across zygosity for same-sex pairs (separating means and variances by sex). d, equating males in opposite-sex pairs with males in same-sex pairs (and the same for females). e, testing for sex differences in means and variances. −2LL, likelihood-ratio test (minus two times the log-likelihood of the data); BIC, Bayesian Information Criteria; AIC, Akaike's Information Criteria*; df,* degrees of freedom; $^{\Delta}\chi^2$, difference in −2LL statistic between models; $^{\Delta}df$, difference in degrees of freedom between models; AIC calculated as $^{\Delta}\chi^2$ minus twice $^{\Delta}df$.

Table S9. *Assumption testing for anhedonia*

					Comparative fit with model in row above				Comparative fit with saturated model			
Model	-2LL	*df*	Parameters	BIC	$^{\Delta}\chi^2$	$^{\Delta}df$	*p*	AIC	$^{\Delta}\chi^2$	$^{\Delta}df$	*p*	AIC
Saturated	43,667.51	16,410	70	-100,155.74	-	-	-	-	-	-	-	-
a	43,668.60	16,418	62	-100,224.75	1.10	8	.99	-14.90	1.10	8	.99	-14.90
b	43,681.07	16,426	54	-100,282.40	12.47	8	.13	-3.53	13.57	16	.63	-18.43
c	43,684.75	16,434	46	-100,348.83	3.68	8	.88	-12.32	17.25	24	.84	-30.75
d	43,696.87	16,442	38	-100,406.83	12.12	8	.15	-3.88	29.37	32	.60	-34.63
e	43,699.35	16,446	34	-100,439.41	2.48	4	.65	-5.52	31.85	36	.67	-40.15

Note. Model definitions: a, equating means within twin pairs for same-sex pairs. b, equating variances within twin pairs for same-sex pairs. c, equating means and variances across zygosity for same-sex pairs (separating means and variances by sex). d, equating males in opposite-sex pairs with males in same-sex pairs (and the same for females). e, testing for sex differences in means and variances. −2LL, likelihood-ratio test (minus two times the log-likelihood of the data); BIC, Bayesian Information Criteria; AIC, Akaike's Information Criteria; *df,* degrees of freedom; $^{\Delta}\chi^2$, difference in −2LL statistic between models; $^{\Delta}df$, difference in degrees of freedom between models; AIC calculated as $^{\Delta}\chi^2$ minus twice $^{\Delta}df$.

Table S10. *Assumption testing for parent-rated negative symptoms*

					Comparative fit with model in row above				Comparative fit with saturated model			
Model	-2LL	*df*	Parameters	BIC	$^{\Delta}\chi^2$	$^{\Delta}df$	*p*	AIC	$^{\Delta}\chi^2$	$^{\Delta}df$	*p*	AIC
Saturated	40,075.14	16,454	70	-104,133.73	-	-	-	-	-	-	-	-
a	40,122.03	16,462	62	-104,156.96	46.89	8	<.001	30.89	46.89	8	<.001	30.89
b	40,131.52	16,470	54	-104,217.59	9.49	8	.30	-6.51	56.38	16	<.001	24.38
c	40,140.88	16,478	46	-104,278.34	9.37	8	.31	-6.63	65.74	24	<.001	17.74
d	40,178.75	16,486	38	-104,310.59	37.86	8	<.001	21.86	103.60	32	<.001	39.60
e	40,205.28	16,490	34	-104,319.11	26.53	4	<.001	18.53	130.14	36	<.001	58.14

Note. Model definitions: a, equating means within twin pairs for same-sex pairs. b, equating variances within twin pairs for same-sex pairs. c, equating means and variances across zygosity for same-sex pairs (separating means and variances by sex). d, equating males in opposite-sex pairs with males in same-sex pairs (and the same for females). e, testing for sex differences in means and variances. −2LL, likelihood-ratio test (minus two times the log-likelihood of the data); BIC, Bayesian Information Criteria; AIC, Akaike's Information Criteria*; df,* degrees of freedom; $^{\Delta}\chi^2$, difference in −2LL statistic between models; $^{\Delta}df$, difference in degrees of freedom between models; AIC calculated as $^{\Delta}\chi^2$ minus twice $^{\Delta}df$.

Table S11. *Twin model statistics for paranoia*

Model	-2LL	*df*	Parameters	BIC	Comparative model	$^{\Delta}\chi^2$	$^{\Delta}df$	*p*	AIC
Saturated	43,244.21	16,398	70	-100,473.86	-	-	-	-	-
Full ACE	43,329.40	16,443	25	-100,783.06	Saturated	85.19	45	<.001	-4.81
Full ADE	43,340.58	16,443	25	-100,771.88	Saturated	96.37	45	<.001	6.37
ACE Quan	43,348.80	16,446	22	-100,789.96	Full ACE	19.40	3	<.001	13.40
ACE Scalar	43,358.18	16,449	19	-100,806.87	ACE Quant	9.38	3	.03	3.38
ACE Hom**	43,364.40	16,455	13	-100,853.24	ACE Scalar	6.22	6	.40	-5.78
Submodels									
AE	43,442.04	16,458	10	-100,801.89	ACE Hom	77.65	3	<.001	71.65
CE	43,467.17	16,458	10	-100,776.77	ACE Hom	102.77	3	<.001	96.77
E	44,303.823	16,461	7	-99,967.00	ACE Hom	938.83	6	<.001	926.83

Note. Full ACE, ACE model with quantitative and qualitative sex differences; Full ADE, ADE model with quantitative and qualitative sex differences; ACE Quan, ACE model with quantitative sex differences; ACE Scalar, ACE model without sex differences on the aetiological correlations; ACE Hom, ACE model without sex differences; −2LL, likelihood-ratio test (minus two times the log-likelihood of the data); BIC, Bayesian Information Criteria; AIC, Akaike's Information Criteria*; df,* degrees of freedom; $^{\Delta}\chi^2$, difference in −2LL statistic between models; $^{\Delta}df$, difference in degrees of freedom between models; AIC calculated as $^{\Delta}\chi^2$ minus twice $^{\Delta}df$; **, best-fitting overall model; Full sex differences models are compared to the saturated model. Subsequent models are compared to and nested within the antecedent model. Submodels are compared to and nested within the best-fitting ACE / ADE model.

Table S12. *Twin model statistics for hallucinations*

Model	-2LL	*df*	Parameters	BIC	Comparative model	$^{\Delta}\chi^2$	$^{\Delta}df$	*p*	AIC
Saturated	43,756.14	16,413	70	-100,093.40	-	-	-	-	-
Full ACE	43,819.93	16,458	25	-100,424.00	Saturated	63.79	45	.03	-26.21
ACE Quan	43,827.65	16,461	22	-100,442.57	Full ACE	7.72	3	.05	1.72
ACE Scalar	43,837.65	16,464	19	-100,458.87	ACE Quant	10.00	3	.02	4.00
ACE Hom**	43,846.91	16,470	13	-100,502.19	ACE Scalar	9.26	6	.16	-2.73
Submodels									
AE	43,933.49	16,473	10	-100,441.91	ACE Hom	86.58	3	<.001	80.58
CE	43,923.37	16,473	10	-100,452.03	ACE Hom	76.46	3	<.001	70.46
E	44,641.98	16,476	7	-99,759.71	ACE Hom	795.07	6	<.001	782.07

Note. Full ACE, ACE model with quantitative and qualitative sex differences; ACE Quan, ACE model with quantitative sex differences; ACE Scalar, ACE model without sex differences on the aetiological correlations; ACE Hom, ACE model without sex differences; −2LL, likelihood-ratio test (minus two times the log-likelihood of the data); BIC, Bayesian Information Criteria; AIC, Akaike's Information Criteria; *df,* degrees of freedom; $^{\Delta}\chi^2$, difference in −2LL statistic between models; $^{\Delta}df$, difference in degrees of freedom between models; AIC calculated as $^{\Delta}\chi^2$ minus twice $^{\Delta}df$; **, best-fitting overall model; Full sex differences models are compared to the saturated model. Subsequent models are compared to and nested within the antecedent model. Submodels are compared to and nested within the best-fitting ACE model.

Table S13. *Twin model statistics for cognitive disorganization*

Model	-2LL	*df*	Parameters	BIC	Comparative model	$^{\Delta}\chi^2$	$^{\Delta}df$	*p*	AIC
Saturated	42,957.75	16,401	70	-100,786.61	-	-	-	-	-
Full ACE	43,037.59	16,446	25	-101,101.17	Saturated	79.84	45	<.001	-10.16
Full ADE	43,041.44	16,446	25	-101,097.30	Saturated	83.69	45	<.00	-6.31
ACE Quan	43,052.23	16,449	22	-101,112.83	Full ACE	14.64	3	<.001	8.64
ACE Scalar	43,059.55	16,452	19	-101,131.80	ACE Quant	7.32	3	.06	1.32
ACE Hom**	43,066.06	16,458	13	-101,177.87	ACE Scalar	6.51	6	.37	-5.78
Submodels									
AE	43,117.87	16,461	10	-101,152.79	ACE Hom	51.81	3	<.001	45.38
CE	43,166.67	16,461	10	-101,103.56	ACE Hom	100.61	3	<.001	94.61
E	43,743.21	16,464	7	-100,553.31	ACE Hom	677.15	6	<.001	665.15

Note. Full ACE, ACE model with quantitative and qualitative sex differences; Full ADE, ADE model with quantitative and qualitative sex differences; ACE Quan, ACE model with quantitative sex differences; ACE Scalar, ACE model without sex differences on the aetiological correlations; ACE Hom, ACE model without sex differences; −2LL, likelihood-ratio test (minus two times the log-likelihood of the data); BIC, Bayesian Information Criteria; AIC, Akaike's Information Criteria*; df,* degrees of freedom; $^{\Delta}\chi^2$, difference in −2LL statistic between models; $^{\Delta}df$, difference in degrees of freedom between models; AIC calculated as $^{\Delta}\chi^2$ minus twice $^{\Delta}df$; **, best-fitting overall model; Full sex differences models are compared to the saturated model. Subsequent models are compared to and nested within the antecedent model. Submodels are compared to and nested within the best-fitting ACE / ADE model.

Table S14. *Twin model statistics for grandiosity*

Model	-2LL	*df*	Parameters	BIC	Comparative model	$^{\Delta}\chi^2$	$^{\Delta}df$	*p*	AIC
Saturated	43,969.74	16,402	70	-99,783.39	-	-	-	-	-
Full ACE	44,017.11	16,447	25	-100,130.41	Saturated	47.37	45	.38	-42.63
Full ADE	44,044.02	16,447	25	-100,103.50	Saturated	74.28	45	<.001	-15.72
ACE Quan	44,036.19	16,450	22	-100,137.63	Full ACE	19.08	3	<.001	13.08
ACE Scalar	43,041.75	16,453	19	-100,158.36	ACE Quant	5.56	3	.14	-0.44
ACE Hom**	44,057.15	16,459	13	-100,195.55	ACE Scalar	15.40	6	.02	3.40
Submodels									
AE	44,140.66	16,462	10	-100,138.33	ACE Hom	83.51	3	<.001	77.51
CE	44,141.66	16,462	10	-100,137.33	ACE Hom	84.51	3	<.001	78.51
E	44,887.09	16,465	7	-99,418.19	ACE Hom	829.94	6	<.001	817.94

Note. Full ACE, ACE model with quantitative and qualitative sex differences; Full ADE, ADE model with quantitative and qualitative sex differences; ACE Quan, ACE model with quantitative sex differences; ACE Scalar, ACE model without sex differences on the aetiological correlations; ACE Hom, ACE model without sex differences; −2LL, likelihood-ratio test (minus two times the log-likelihood of the data); BIC, Bayesian Information Criteria; AIC, Akaike's Information Criteria*; df,* degrees of freedom; $^{\Delta}\chi^2$, difference in −2LL statistic between models; $^{\Delta}df$, difference in degrees of freedom between models; AIC calculated as $^{\Delta}\chi^2$ minus twice $^{\Delta}df$; **, best-fitting overall model; Full sex differences models are compared to the saturated model. Subsequent models are compared to and nested within the antecedent model. Submodels are compared to and nested within the best-fitting ACE / ADE model.

Table S15. *Twin model statistics for anhedonia*

Model	-2LL	*df*	Parameters	BIC	Comparative model	$^{\Delta}\chi^2$	$^{\Delta}df$	*p*	AIC
Saturated	43,667.51	16,410	70	-100,155.74	-	-	-	-	-
Full ACE	43,741.63	16,455	25	-100,476.00	Saturated	74.12	45	<.001	-15.88
Full ADE	44,723.41	16,455	25	-100,494.23	Saturated	55.91	45	.13	-34.09
ADE Quan	43,735.51	16,458	22	-100,508.42	Full ADE	12.10	3	.01	6.10
ADE Scalar	43,743.33	16,461	19	-100,526.89	ADE Quant	7.82	3	.05	1.82
ADE Hom	43,750.27	16,467	13	-100,572.54	ADE Scalar	6.94	6	.33	-5.06
Submodels									
AE**	43,785.25	16,470	10	-100,563.86	ADE Hom	34.98	3	<.001	28.98
E	44,457.19	16,473	7	-99,918.21	ADE Hom	706.92	6	<.001	694.92

Note. Full ACE, ACE model with quantitative and qualitative sex differences; Full ADE, ADE model with quantitative and qualitative sex differences; ACE Quan, ACE model with quantitative sex differences; ACE Scalar, ACE model without sex differences on the aetiological correlations; ACE Hom, ACE model without sex differences; −2LL, likelihood-ratio test (minus two times the log-likelihood of the data); BIC, Bayesian Information Criteria; AIC, Akaike's Information Criteria; *df,* degrees of freedom; $^{\Delta}\chi^2$, difference in −2LL statistic between models; $^{\Delta}df$, difference in degrees of freedom between models; AIC calculated as $^{\Delta}\chi^2$ minus twice $^{\Delta}df$; **, best-fitting overall model; Full sex differences models are compared to the saturated model. Subsequent models are compared to and nested within the antecedent model. Submodels are compared to and nested within the best-fitting ACE / ADE model.

Table S16. *Twin model statistics for parent-rated negative symptoms*

Model	-2LL	*df*	Parameters	BIC	Comparative model	$^{\Delta}\chi^2$	$^{\Delta}df$	*p*	AIC
Saturated	40,075.14	16,454	70	-104,133.73	-	-	-	-	-
Full ACE	40,187.65	16,499	25	-104,415.62	Saturated	112.51	45	<.001	22.51
ACE Quan	40,268.73	16,502	22	-104,360.83	Full ACE	81.08	3	<.001	75.08
ACE Scalar	40,268.85	16,505	19	-104,387.01	ACE Quant	0.12	3	.99	-5.88
ACE Hom**	40,283.92	16,511	13	-104,424.53	ACE Scalar	15.07	6	.02	3.07
Submodels									
AE	40,867.24	16,514	10	-103,867.49	ACE Hom	583.32	3	<.001	577.32
CE	40,887.89	16,514	10	-103,846.85	ACE Hom	603.97	3	<.001	597.97
E	44,531.44	16,517	7	-100,229.59	ACE Hom	4,247.52	6	<.001	4235.52

Note. Full ACE, ACE model with quantitative and qualitative sex differences; ACE Quan, ACE model with quantitative sex differences; ACE Scalar, ACE model without sex differences on the aetiological correlations; ACE Hom, ACE model without sex differences; −2LL, likelihood-ratio test (minus two times the log-likelihood of the data); BIC, Bayesian Information Criteria; AIC, Akaike's Information Criteria; *df,* degrees of freedom; $^{\Delta}\chi^2$, difference in −2LL statistic between models; $^{\Delta}df$, difference in degrees of freedom between models; AIC calculated as $^{\Delta}\chi^2$ minus twice $^{\Delta}df$; **, best-fitting overall model; Full sex differences models are compared to the saturated model. Subsequent models are compared to and nested within the antecedent model. Submodels are compared to and nested within the best-fitting ACE model.

Table S17. *Cholesky estimates*

	Total shared	Total unique	Shared A	Unique A	Shared C	Unique C	Shared E	Unique E
Paranoia	.44	.56	.25	.08	.08	.04	.11	.44
Hallucinations	.46	.54	.25	.08	.12	.04	.09	.42
Cognitive disorganization	.58	.43	.38	.00	.06	.00	.14	.43
Grandiosity	.44	.57	.26	.00	.11	.08	.07	.49
Anhedonia	.42	.58	.32	.13	-	-	.10	.44
PRNS	.43	.57	.27	.18	.13	.25	.03	.14

Note. A, Additive genetic effects; C, Common environmental effects; E, Nonshared environmental effects; PRNS, Parent-rated negative symptoms; Cholesky estimates represent the proportion of variation at time 2 that is due to influences shared with time 1, and those that are unique to time 2.

Table S18. *Genetic and environmental correlations for best-fitting bivariate models*

	*r*A	*r*C	*r*E
Paranoia	.87 [.73, 1.00]	.83 [.64, 1.00]	.45 [.39, .50]
Hallucinations	.87 [.70, 1.00]	.87 [.71, 1.00]	.41 [.35, .46]
Cognitive disorganization	1.00 [.92, 1.00]	1.00 [.84, 1.00]	.49 [.45, .53]
Grandiosity	1.00 [.84, 1.00]	.76 [.63, .91]	.36 [.31, .41]
Anhedonia	.84 [.79, .90]	-	.44 [.39, .49]
PRNS	.77 [.72, .83]	.59 [.52, .65]	.45 [.38, .51]

Note. *r*A, Genetic correlation; *r*C, Common environment correlation; *r*E, Nonshared environment correlation; PRNS, Parent-rated negative symptoms; 95% CI in parentheses.

Table S19. *Descriptives and mean differences for psychotic experiences and negative symptoms subscales at time 1 by group*

	Group			
	Low-scoring (LS)	Increasing (I)	Decreasing (D)	Persistent (P)
Paranoia				
N (%)	1,215 (84.7%)	64 (4.5%)	77 (5.4%)	79 (5.5%)
Paranoia mean score (*SD*)	11.09 (8.85)	22.19 (9.71)	42.87 (5.89)	45.76 (8.17)
Cohen's *d* [95% CI]	LS versus I = 1.25 [0.99, 1.50]		D versus P = 0.41 [0.09, 0.72]	
Hallucinations				
N (%)	1,182 (82.1%)	86 (6.0%)	92 (6.4%)	79 (5.5%)
Hallucinations mean score (*SD*)	3.61 (4.21)	10.49 (4.99)	22.57 (5.15)	24.23 (5.68)
Cohen's *d* [95% CI]	LS versus I = 1.61 [1.39, 1.84]		D versus P = 0.31 [0.01, 0.61]	
Cognitive disorganization				
N (%)	1,138 (79.1%)	79 (5.5%)	105 (7.3%)	116 (8.1%)
Cognitive disorganization mean score (*SD*)	3.42 (2.37)	6.45 (1.74)	9.67 (0.70)	10.07 (0.74)
Cohen's *d* [95% CI]	LS versus I = 1.30 [1.06, 1.53]		D versus P = 0.56 [0.29, 0.82]	
Grandiosity				
N (%)	1,212 (84.3%)	61 (4.3%)	72 (5.0%)	92 (6.4%)
Grandiosity mean score (*SD*)	3.96 (3.14)	7.36 (3.30)	16.81 (2.93)	17.57 (2.81)
Cohen's *d* [95% CI]	LS versus I = 1.08 [0.82, 1.34]		D versus P = 0.27 [-0.04, 0.58]	

Anhedonia				
N (%)	1,141 (83.5%)	81 (5.9%)	63 (4.6%)	82 (6.0%)
Anhedonia mean score (*SD*)	14.25 (6.39)	22.05 (5.66)	34.22 (2.83)	35.74 (3.17)
Cohen's *d* [95% CI]	LS versus I = 1.23 [1.00, 1.46]		D versus P = 0.50 [0.17, 0.84]	
PRNS				
N (%)	1,173 (81.9%)	97 (6.8%)	66 (4.6%)	96 (6.7%)
PRNS mean score (*SD*)	1.91 (2.35)	5.48 (3.03)	14.69 (3.55)	16.10 (4.05)
Cohen's *d* [95% CI]	LS versus I = 1.48 [1.27, 1.70])		D versus P = 0.37 [0.05, 0.68]	

Note. N, Number of individuals; PRNS, Parent-rated negative symptoms; LS, Low-scoring group; I, Increasing group; D, Decreasing group; P, Persistent group. Cohen's *d* measure of strength of association for unequal sample sizes (effect size of the standardized difference between the means; equal to the difference between the means divided by pooled *SD*); One randomly selected twin per pair included in analyses.

Table S20. *Mean differences for depression traits at time 1 by group*

	Group			
	Low-scoring (LS)	Increasing (I)	Decreasing (D)	Persistent (P)
Paranoia				
N (%)	1,214 (84.7%)	64 (4.5%)	77 (5.4%)	79 (5.5%)
SMFQ mean score (*SD*)	3.89 (4.67)	7.92 (6.44)	9.88 (5.49)	12.01 (7.24)
Cohen's *d* [95% CI]	LS versus I = 0.84 [0.59, 1.10])	I versus P = 0.59 [0.26, 0.93]	D versus P = 0.33 [0.02, 0.65]	LS versus P = 1.67 [1.43, 1.91]
Hallucinations				
N (%)	1,181 (82.1%)	86 (6.0%)	92 (6.4%)	79 (5.5%)
SMFQ mean score (*SD*)	4.03 (4.95)	8.11 (6.69)	7.89 (5.41)	9.69 (6.65)
Cohen's *d* [95% CI]	LS versus I = 0.80 [0.58, 1.02]	I versus P = 0.24 [-0.07, 0.54]	D versus P = 0.30 [-0.01, 0.60]	LS versus P = 1.12 [0.88, 1.35]
Cognitive disorganization				
N (%)	1,137 (79.1%)	79 (5.5%)	105 (7.3%)	116 (8.1%)
SMFQ mean score (*SD*)	3.53 (4.34)	7.83 (6.56)	9.68 (5.84)	11.21 (6.62)
Cohen's *d* [95% CI]	LS versus I = 0.95 [0.72, 1.18]	I versus P = 0.51 [0.22, 0.80]	D versus P = 0.24 [-0.02, 0.51]	LS versus P = 1.67 [1.47, 1.87]
Grandiosity				
N (%)	1,211 (84.3%)	61 (4.2%)	72 (5.0%)	92 (6.4%)
SMFQ mean score (*SD*)	4.86 (5.51)	4.78 (5.43)	4.97 (5.45)	4.41 (5.38)

Cohen's *d* [95% CI]	LS versus I = -0.02 [-0.27, 0.24]	I versus P = 0.06 [-0.39, 0.23]	D versus P = 0.10 [-0.41, 0.21]	LS versus P = -0.08 [-0.29, 0.13]
Anhedonia				
N (%)	1,140 (83.5%)	81 (5.9%)	63 (4.6%)	82 (6.0%)
SMFQ mean score (*SD*)	4.53 (5.22)	5.22 (5.17)	6.39 (6.78)	7.05 (6.82)
Cohen's *d* [95% CI]	LS versus I = 0.13 [-0.09, 0.36]	I versus P = 0.30 [-0.01, 0.61]	D versus P = 0.10 [-0.23, 0.43]	LS versus P = 0.47 [0.25, 0.70]
PRNS				
N (%)	1,172 (81.9%)	97 (6.8%)	66 (4.6%)	96 (6.7%)
SMFQ mean score (*SD*)	4.46 (5.35)	6.49 (5.70)	5.86 (5.23)	6.99 (6.13)
Cohen's *d* [95% CI]	LS versus I = 0.38 [0.17, 0.59]	I versus P = 0.08 [-0.20, 0.37]	D versus P = 0.20 [-0.12, 0.51]	LS versus P = 0.47 [0.26, 0.68]

Note. N, Number of individuals; PRNS, Parent-rated negative symptoms; SMFQ, Short Mood and Feeling Questionnaire (self-rated) measure of depression traits; LS, Low-scoring group; I, Increasing group; D, Decreasing group; P, Persistent group. Cohen's *d* measure of strength of association for unequal sample sizes (effect size of the standardized difference between the means; equal to the difference between the means divided by pooled *SD*); One randomly selected twin per pair included in analyses; SMFQ score at time 1 for whole sample: Mean = 4.83; *SD* = 5.49 (*N* = 1,447).

Table S21. *Mean differences for emotional problems at time 1 by group*

	Group			
	Low-scoring (LS)	Increasing (I)	Decreasing (D)	Persistent (P)
Paranoia				
N (%)	1,213 (84.8%)	63 (4.4%)	77 (5.4%)	78 (5.5%)
SDQ mean score (*SD*)	2.81 (2.26)	4.22 (2.61)	4.95 (2.47)	5.85 (2.59)
Cohen's *d* [95% CI]	LS versus I = 0.62 [0.36, 0.87]	I versus P = 0.63 [0.29, 0.97]	D versus P = 0.36 [0.04, 0.67]	LS versus P = 1.33 [1.10, 1.57]
Hallucinations				
N (%)	1,179 (82.2%)	86 (6.0%)	92 (6.4%)	78 (5.4%)
SDQ mean score (*SD*)	2.85 (2.30)	4.28 (2.81)	4.16 (2.36)	5.31 (2.61)
Cohen's *d* [95% CI]	LS versus I = 0.61 [0.39, 0.83]	I versus P = 0.38 [0.07, 0.69]	D versus P = 0.46 [0.16, 0.77]	LS versus P = 1.06 [0.83, 1.29]
Cognitive disorganization				
N (%)	1,135 (79.1%)	79 (5.5%)	105 (7.3%)	115 (8.0%)
SDQ mean score (*SD*)	2.61 (2.19)	4.23 (2.14)	5.31 (2.32)	5.82 (2.14)
Cohen's *d* [95% CI]	LS versus I = 0.74 [0.51, 0.97]	I versus P = 0.74 [0.45, 1.04]	D versus P = 0.21 [-0.06, 0.47]	LS versus P = 1.47 [1.27, 1.67]
Grandiosity				
N (%)	1,209 (84.4%)	60 (4.2%)	72 (5.0%)	92 (6.4%)
SDQ mean score (*SD*)	3.20 (2.45)	3.08 (2.23)	3.03 (2.52)	2.64 (2.48)
Cohen's *d* [95% CI]	LS versus I = -0.05 [-0.31, 0.21]	I versus P = 0.19 [-0.51, 0.14]	D versus P = -0.16 [-0.47, 0.15]	LS versus P = -0.23 [-0.44, -0.02]

Anhedonia				
N (%)	1,139 (83.5%)	81 (5.9%)	62 (4.6%)	82 (6.0%)
SDQ mean score (*SD*)	3.11 (2.42)	3.25 (2.48)	3.59 (2.70)	3.45 (2.62)
Cohen's *d* [95% CI]	LS versus I = 0.06 [-0.17, 0.28]	I versus P = 0.07 [-0.23, 0.39]	D versus P = 0.05 [-0.38, 0.28]	LS versus P = 0.14 [-0.09, 0.36]
PRNS				
N (%)	1,170 (81.9%)	97 (6.8%)	65 (4.6%)	96 (6.7%)
SDQ mean score (*SD*)	3.01 (2.44)	3.47 (2.28)	3.72 (2.30)	4.17 (2.52)
Cohen's *d* [95% CI]	LS versus I = 0.19 [-0.02, 0.40]	I versus P = 0.29 [0.01, 0.58]	D versus P = 0.19 [-0.13, 0.50]	LS versus P = 0.47 [0.27, 0.68]

Note. N, Number of individuals; PRNS, Parent-rated negative symptoms; SDQ emotional problems, Strengths and Difficulty Questionnaire emotional problems subscale (self-rated); LS, Low-scoring group; I, Increasing group; D, Decreasing group; P, Persistent group. Cohen's *d* measure of strength of association for unequal sample sizes (effect size of the standardized difference between the means; equal to the difference between the means divided by pooled *SD*); One randomly selected twin per pair included in analyses; SDQ emotional problems score at time 1 for whole sample: Mean = 3.16; *SD* = 2.45 (*N* = 1,444).

Table S22. *Mean differences for conduct problems at time 1 by group*

	Group			
	Low-scoring (LS)	Increasing (I)	Decreasing (D)	Persistent (P)
Paranoia				
N (%)	1,213 (84.7%)	63 (4.5%)	77 (5.4%)	78 (5.5%)
SDQ mean score (*SD*)	1.63 (1.48)	2.94 (1.92)	2.70 (1.71)	3.18 (1.78)
Cohen's *d* [95% CI]	LS versus I = 0.76 [0.65, 0.88])	I versus P = 0.13 [-0.20, 0.46]	D versus P = 0.28 [-0.04, 0.59]	LS versus P = 0.95 [0.83, 1.06]
Hallucinations				
N (%)	1,179 (82.1%)	86 (6.0%)	92 (6.4%)	78 (5.5%)
SDQ mean score (*SD*)	1.61 (1.44)	3.01 (1.84)	2.79 (1.88)	2.71 (2.02)
Cohen's *d* [95% CI]	LS versus I = 0.85 [0.73, 0.96]	I versus P = -0.16 [-0.46, 0.15]	D versus P = -0.04 [-0.34, 0.26]	LS versus P = 0.63 [0.51, 0.74]
Cognitive disorganization				
N (%)	1,135 (79.1%)	79 (5.5%)	105 (7.3%)	115 (8.1%)
SDQ mean score (*SD*)	1.58 (1.44)	2.25 (1.60)	3.02 (1.60)	2.95 (1.68)
Cohen's *d* [95% CI]	LS versus I = 0.44 [0.33, 0.55]	I versus P = 0.43 [0.14, 0.71]	D versus P = -0.04 [-0.31, 0.22]	LS versus P = 0.88 [0.76, 0.99]
Grandiosity				
N (%)	1,209 (84.3%)	60 (4.2%)	72 (5.0%)	92 (6.4%)
SDQ mean score (*SD*)	1.76 (1.55)	2.22 (1.76)	2.35 (2.06)	2.20 (1.65)
Cohen's *d* [95% CI]	LS versus I = 0.28 [0.17 ,0.39]	I versus P = -0.01 [-0.33, 0.31]	D versus P = -0.08 [-0.39, 0.23]	LS versus P = 0.28 [0.17, 0.38]

Anhedonia				
N (%)	1,139 (83.5%)	81 (5.9%)	62 (4.6%)	82 (6.0%)
SDQ mean score (*SD*)	1.77 (1.55)	2.06 (1.84)	2.03 (1.59)	2.38 (1.90)
Cohen's *d* [95% CI]	LS versus I = 0.17 [0.06, 0.28]	I versus P = 0.17 [-0.14, 0.48]	D versus P = 0.20 [-0.13, 0.53]	LS versus P = 0.35 [0.24, 0.47]
PRNS				
N (%)	1,170 (81.9%)	97 (6.8%)	65 (4.6%)	96 (6.7%)
SDQ mean score (*SD*)	1.71 (1.54)	2.06 (1.59)	2.53 (1.94)	2.65 (1.84)
Cohen's *d* [95% CI]	LS versus I = 0.22 [0.11, 0.33]	I versus P = 0.34 [0.06, 0.63]	D versus P = 0.06 [-0.25, 0.37]	LS versus P = 0.55 [0.44, 0.68]

Note. N, Number of individuals; PRNS, Parent-rated negative symptoms; SDQ conduct problems, Strengths and Difficulty Questionnaire conduct problems subscale (self-rated); LS, Low-scoring group; I, Increasing group; D, Decreasing group; P, Persistent group. Cohen's *d* measure of strength of association for unequal sample sizes (effect size of the standardized difference between the means; equal to the difference between the means divided by pooled *SD*); One randomly selected twin per pair included in analyses.

Table S23. *Mean differences for hyperactivity at time 1 by group*

	Group			
	Low-scoring (LS)	Increasing (I)	Decreasing (D)	Persistent (P)
Paranoia				
N (%)	1,213 (84.7%)	63 (4.5%)	77 (5.4%)	78 (5.5%)
SDQ mean score (*SD*)	3.62 (2.45)	5.10 (2.51)	5.11 (2.30)	5.46 (2.39)
Cohen's *d* [95% CI]	LS versus I = 0.60 [0.49, 0.71])	I versus P = 0.15 [-0.16, 0.45]	D versus P = 0.15 [-0.17, 0.47]	LS versus P = 0.76 [0.65, 0.87]
Hallucinations				
N (%)	1,179 (82.1%)	86 (6.0%)	92 (6.4%)	78 (5.5%)
SDQ mean score (*SD*)	3.59 (2.43)	5.01 (2.45)	5.17 (2.39)	5.17 (2.70)
Cohen's *d* [95% CI]	LS versus I = 0.58 [0.47, 0.70]	I versus P = 0.06 [-0.24, 0.37]	D versus P = 0.00 [-0.30, 0.30]	LS versus P = 0.62 [0.50, 0.73]
Cognitive disorganization				
N (%)	1,135 (79.1%)	79 (5.5%)	105 (7.3%)	115 (8.1%)
SDQ mean score (*SD*)	3.31 (2.26)	5.13 (2.24)	5.97 (2.22)	6.59 (2.12)
Cohen's *d* [95% CI]	LS versus I = 0.81 [0.69, 0.92]	I versus P = 0.67 [0.38, 0.96]	D versus P = 0.29 [0.02, 0.55]	LS versus P = 1.50 [1.37, 1.62]
Grandiosity				
N (%)	1,209 (84.3%)	60 (4.2%)	72 (5.0%)	92 (6.4%)
SDQ mean score (*SD*)	3.82 (2.46)	3.96 (2.35)	4.47 (2.90)	3.90 (2.81)
Cohen's *d* [95% CI]	LS versus I = 0.06 [-0.05 ,0.17]	I versus P = -0.02 [-0.34, 0.30]	D versus P = -0.20 [-0.51, 0.11]	LS versus P = 0.03 [-0.08, 0.14]

Anhedonia				
N (%)	1,139 (83.5%)	81 (5.9%)	62 (4.6%)	82 (6.0%)
SDQ mean score (*SD*)	3.77 (2.47)	4.76 (2.60)	3.84 (2.67)	4.11 (2.42)
Cohen's *d* [95% CI]	LS versus I = 0.39 [0.28, 0.50]	I versus P = -0.26 [-0.57, 0.]	D versus P = 0.11 [-0.22, 0.43]	LS versus P = 0.14 [0.03, 0.25]
PRNS				
N (%)	1,170 (81.9%)	97 (6.8%)	65 (4.6%)	96 (6.7%)
SDQ mean score (*SD*)	3.69 (2.50)	4.42 (2.52)	4.74 (2.24)	4.83 (2.51)
Cohen's *d* [95% CI]	LS versus I = 0.29 [0.18, 0.40]	I versus P = 0.16 [-0.12, 0.45]	D versus P = 0.04 [-0.27, 0.35]	LS versus P = 0.46 [0.34, 0.57]

Note. N, Number of individuals; PRNS, Parent-rated negative symptoms; SDQ hyperactivity, Strengths and Difficulty Questionnaire hyperactivity subscale (self-rated); LS, Low-scoring group; I, Increasing group; D, Decreasing group; P, Persistent group. Cohen's *d* measure of strength of association for unequal sample sizes (effect size of the standardized difference between the means; equal to the difference between the means divided by pooled *SD*); One randomly selected twin per pair included in analyses.

Table S24 *Mean differences for peer problems at time 1 by group*

	Group			
	Low-scoring (LS)	Increasing (I)	Decreasing (D)	Persistent (P)
Paranoia				
N (%)	1,214 (84.7%)	63 (4.5%)	77 (5.4%)	78 (5.5%)
SDQ mean score (*SD*)	1.60 (1.54)	2.52 (1.97)	3.46 (2.11)	3.99 (2.31)
Cohen's *d* [95% CI]	LS versus I = 0.52 [0.44, 0.60])	I versus P = 0.69 [0.35-1.02]	D versus P = 0.24 [-0.08, 0.56]	LS versus P = 1.22 [1.10, 1.34]
Hallucinations				
N (%)	1,179 (82.1%)	86 (6.0%)	92 (6.4%)	78 (5.5%)
SDQ mean score (*SD*)	1.73 (1.73)	2.24 (1.90)	2.43 (1.87)	2.82 (1.87)
Cohen's *d* [95% CI]	LS versus I = 0.28 [0.17, 0.39]	I versus P = 0.31 [0.00, 0.62]	D versus P = 0.21 [-0.09, 0.51]	LS versus P = 0.61 [0.49, 0.72]
Cognitive disorganization				
N (%)	1,135 (79.1%)	79 (5.5%)	105 (7.3%)	115 (8.1%)
SDQ mean score (*SD*)	1.63 (1.64)	2.40 (1.91)	2.65 (1.98)	3.11 (2.03)
Cohen's *d* [95% CI]	LS versus I = 0.43 [0.32, 0.55]	I versus P = 0.36 [0.08, 0.64]	D versus P = 0.23 [-0.04, 0.50]	LS versus P = 0.80 [0.69-0.92]
Grandiosity				
N (%)	1,209 (84.3%)	60 (4.2%)	72 (5.0%)	92 (6.4%)
SDQ mean score (*SD*)	1.84 (1.73)	2.03 (1.88)	2.24 (2.24)	1.89 (1.96)
Cohen's *d* [95% CI]	LS versus I = 0.11 [-0.01 ,0.22]	I versus P = -0.07 [-0.39, 0.25]	D versus P = -0.17 [-0.47, 0.14]	LS versus P = 0.03 [-0.08, 0.14]

Anhedonia				
N (%)	1,139 (83.5%)	81 (5.9%)	62 (4.6%)	82 (6.0%)
SDQ mean score (*SD*)	1.74 (1.72)	2.32 (2.07)	2.37 (1.67)	2.57 (1.76)
Cohen's *d* [95% CI]	LS versus I = 0.31 [0.19, 0.42]	I versus P = 0.13 [-0.18, 0.44]	D versus P = 0.12 [-0.21, 0.44]	LS versus P = 0.48 [0.36, 0.59]
PRNS				
N (%)	1,170 (81.9%)	97 (6.8%)	65 (4.6%)	96 (6.7%)
SDQ mean score (*SD*)	1.70 (1.72)	2.73 (1.92)	2.38 (1.80)	2.69 (1.85)
Cohen's *d* [95% CI]	LS versus I = 0.57 [0.45, 0.68]	I versus P = -0.02 [-0.31, 0.26]	D versus P = 0.17 [-0.14, 0.48]	LS versus P = 0.55 [0.44, 0.67]

Note. N, Number of individuals; PRNS, Parent-rated negative symptoms; SDQ peer problems, Strengths and Difficulty Questionnaire peer problems subscale (self-rated); LS, Low-scoring group; I, Increasing group; D, Decreasing group; P, Persistent group. Cohen's *d* measure of strength of association for unequal sample sizes (effect size of the standardized difference between the means; equal to the difference between the means divided by pooled *SD*); One randomly selected twin per pair included in analyses.

Table S25. *Correlations for psychotic experiences and negative symptoms with depression traits and SDQ emotional problems and other SDQ subscales*

	Paranoia	Hallucinations	Cognitive disorganization	Grandiosity	Anhedonia	PRNS
SMFQ	.59 (<.001)	.48 (<.001)	.67 (<.001)	.04 (.11)	.17 (<.001)	.27 (<.001)
SDQ emotional problems	.46 (<.001)	.38 (<.001)	.62 (<.001)	-.03 (.24)	.09 (.001)	.25 (<.001)
SDQ conduct problems	.40 (<.001)	.35 (<.001)	.44 (<.001)	.12 (<.001)	.13 (<.001)	.25 (<.001)
SDQ hyperactivity	.32 (<.001)	.31 (<.001)	.61 (<.001)	.06 (p = .03)	.06 (p = .02)	.20 (<.001)
SDQ peer problems	.50 (<.001)	.27 (<.001)	.36 (<.001)	.04 (p = .18)	.24 (<.001)	.26 (<.001)

Note. PRNS, Parent-rated negative symptoms; SMFQ, Short Mood and Feeling Questionnaire (self-rated) measure of depression traits; SDQ, Strengths and Difficulty Questionnaire emotional problems subscale, conduct problems subscale, hyperactivity subscale and peer problems subscale (self-rated); One randomly selected twin per pair included in analyses; Spearman's rho correlation coefficients are reported owing to the skewed distributions of each variable. Pearson's correlation coefficients are reported for SDQ and cognitive disorganization, and SDQ and anhedonia owing to the non-skewed distributions of either variable. p values in parentheses.

Table S26. *Descriptive statistics for depression traits and psychopathology subscales*

	SMFQ	SDQ Emotional problems	SDQ Conduct problems	SDQ Hyperactivity	SDQ Peer problems
N	1,447	1,444	1,444	1,444	1,444
**Missing**	1	4	4	4	4
**Scale Range**	0-26	0-10	0-10	0-10	0-10
**Observed Mean**	4.83	3.16	1.84	3.87	1.87
**95% CI**	4.55-5.12	3.03-3.28	1.76-1.92	3.74-4.00	1.77-1.96
**Median**	3	3	1	4	1
**Mode**	0	0	1	2	1
SD	5.49	2.45	1.60	2.51	1.78
**Variance**	30.15	6.00	2.57	6.30	3.16
**Skewness**	1.51	0.60	1.12	0.39	1.21
**Kurtosis**	1.89	-0.36	1.33	-0.55	1.57
**Cronbach's α**	.92	.74	.59	.77	.63

Note. SMFQ, Short Mood and Feelings Questionnaire; SDQ, Strengths and Difficulties Questionnaire.
