# Supplementary material for: Genetic and environmental influences on the stability of psychotic experiences and negative symptoms in adolescence
Source: J Child Psychol Psychiatry. 2019 Apr 7;60(7):784–92. doi: 10.1111/jcpp.13045 (PMC6619355; doi:10.1111/jcpp.13045)
Supplement: Supplementary file 2 [file JCPP-60-784-s002.docx]

**This is supplementary information for ‘Genetic and environmental influences on the stability of psychotic experiences and negative symptoms in adolescence’ by Laura Havers et al.**

**Appendix S1*.*** *Study details*

Initially TEDS contacted 16,810 families when the twins were 18 months old, of which 13,694 families responded. In sum, 10,868 families were invited to participate in LEAP. Families were not invited either because of study withdrawal, non-return of data, contact difficulties, or medical exclusion. From this, 5,059 twin pairs and 5,076 parents returned data. For LEAP phase 2, 1,773 families were invited and 1,464 families returned data. In the current study, 5,163 twin pairs had LEAP data available, of which 293 pairs were excluded due to non-consent, unknown zygosity, perinatal complications, severe medical disorder, or an autism diagnosis. For the phenotypic stability analyses, participants with data at both ages were included (*N* = 1,448). For the twin model-fitting analyses, *N* = 4,870 twin pairs at time 1 and *N* = 1,464 pairs at time 2 were included in the analyses.

Families were rewarded for their participation in these stages of data collection. Consent from parents and twins was required separately at age 16.

**Appendix S2*.*** *The Specific Psychotic Experiences Questionnaire (SPEQ; Ronald et al., 2014)*

SPEQ subscales were developed from adult scales, through rewording and selection of items. Age-appropriateness and content validity was established through expert clinical consultation and piloting (Ronald et al., 2014). Subscales were derived from Principal Components Analysis. Validity was assessed using family history data and testing agreement with another measure of adolescent PEs (Psychosis-Like Symptoms scale; Zammit, Owen, Evans, Heron, & Lewis, 2011). Differences in the mean scores of singletons and the averaged mean across twin pairs were not significant across PENS except for paranoia (*p* < .05), which was not significant after correcting for multiple testing (*p* < .01) (see Ronald et al., 2014).

The SPEQ is comprised of six subscales: *Paranoia:* Fifteen (15) items measured the frequency of paranoid experiences, for example, “How often have you thought, people might be conspiring against me”? *Hallucinations:* Nine (9) items measured the frequency of hallucinatory experiences, for example, “How often do you hear sounds or music that people near you don’t hear”? *Cognitive disorganization:* Eleven (11) items measured the presence of experiences over the past month, for example, “Are you easily confused if too much happens at the same time”? *Grandiosity:* Eight (8) items measured the frequency of grandiose experiences, for example, “I have special abilities that others do not”. *Hedonia:* Ten (10) items measured anticipatory pleasure, for example, “When something exciting is coming up in my life, I really look forward to it”. Scores were reversed to give a measure of anhedonia. *Parent-rated negative symptoms (PRNS):* Parents reported on 10 items reflecting negative symptoms in each twin, for example, “He/she usually gives brief, one word replies to questions, even if encouraged to say more”.

**Appendix S3**. *Assumptions testing*

Prior to carrying out bivariate twin analysis, assumptions of the twin design were tested for each individual measure. Twin models assume that the mean value of and variance in a given trait does not differ between twins within a pair or across zygosity. To test these assumptions, we first fitted a baseline fully saturated model in which the means and variances were permitted to differ across individuals within a pair and across zygosity. Each assumption was then tested through three submodels: a) a model that equated means within twin pairs; b) a model that equated variances within twin pairs; and c) a model that equated means and variances across twin order. Significant results indicate a violation of a given assumption. For all measures, except for parent-rated negative symptoms, means and variances could be equated across twin order and zygosity. The violation of these assumptions for parent-rated negative symptoms could arise from the relatively strong skew of the scale, which can lead to unequal means across twin order. In addition, a violation of the assumption of equal variances across zygosity can arise when the manner in which parents rate one twin affects how they rate their co-twin (Neale & Maes, 2004). These effects, referred to as sibling contrast effects, are reported fairly commonly for parent-rated measures of child psychopathology (e.g., Ronald et al., 2008, 2014).

Two additional tests were conducted using submodels of the saturated model. First, it was tested whether males and females within opposite-sex pairs had equal means to males and females within same-sex pairs. It was also tested whether it was possible to equate the means and variances across sexes in same-sex pairs, to test for sex differences. These models generally suggested sex differences, except for grandiosity and anhedonia. These results are shown in tables S5-S10.

**Figure S1**. Bivariate Cholesky decomposition solution (left-hand figure) and correlated factors model (right-hand figure) path diagrams.


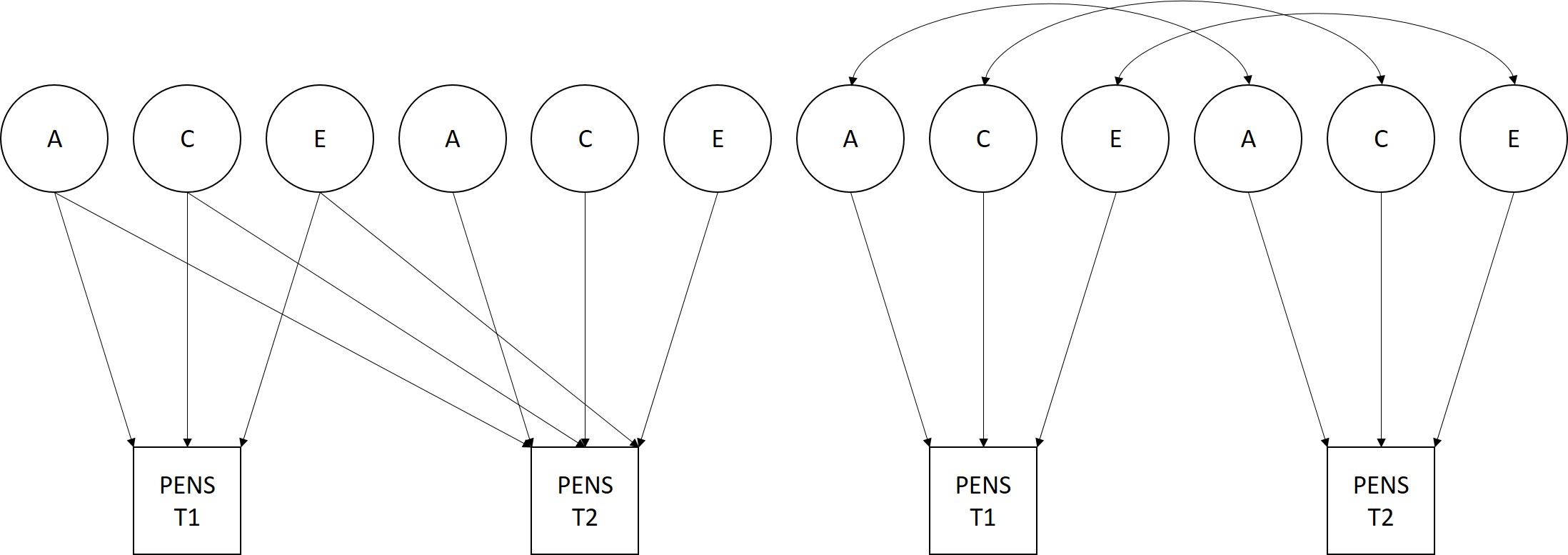


Note. A, Additive genetic influences; C, shared environmental influences; E, nonshared environmental influences; PENS, psychotic experiences and negative symptom traits; T1, time 1; T2, time 2.

**Table S1**. *Frequency and mean differences in demographics of main sample and follow-up sample*

|  | Main sample | Follow-up sample |
| --- | --- | --- |
| General exclusion at 16  Medical exclusion at 16  Perinatal outlier exclusion at first contact  Ethnic origin at first contact (non-white)  Main language spoken at home at first contact (other / English plus other)  Standardised SES composite score at first contact (mean / *SD*) | 315 (6.9%)  170 (3.7%)  97 (2.1%)  311 (6.9%)  147 (3.2%)  0.19 (0.99) | 39 (2.6%)  39 (2.6%)  0 (0.0%)  108 (7.3%)  61 (4.1%)  0.19 (0.99) |

*Note. N* individuals included in analysis: Main sample = 4,535, follow-up sample = 1,487; SES, Socio-economic status; General exclusion applied if no data is available from first contact, for medical exclusion purposes, for perinatal outliers, and for unknown sex or zygosity; Medical exclusion applied if any medical exclusion has been recorded up to and including the age of age of 16.

**Table S2***.* *Descriptives for psychotic experiences and negative symptoms subscales*

|  | Paranoia | | Hallucinations | | Cognitive disorganization | | Grandiosity | | Anhedonia | | PRNS | |
| --- | --- | --- | --- | --- | --- | --- | --- | --- | --- | --- | --- | --- |
|  | Time 1 | Time 2 | Time 1 | Time 2 | Time 1 | Time 2 | Time 1 | Time 2 | Time 1 | Time 2 | Time 1 | Time 2 |
| *N*  Missing  Scale range  Observed  Mean  95% CI  Median  Mode  *SD*  Variance  Skewness  Kurtosis  Cronbach's α | 1,446 2  0-75  0-65  15.21  [14.51,  15.91]  11.00  0  13.60  184.82  1.18  .78  .94 | 1,437  11  0-75  0-72  14.80  [14.09,  15.52]  11.00  0  13.81  190.65  1.40  1.88  .95 | 1,448  0  0-45  0-45  6.39  [5.98, 6.79] 3.00  0  7.82  61.11  1.56  2.15  .91 | 1,439  9  0-45  0-45  6.78  [6.39,  7.17]  4.00  0  7.54  56.87  1.56  2.54  .90 | 1,446  2  0-11  0-11  4.58  [4.42, 4.75]  4.00  4  3.21  10.29  .34  -.96  .82 | 1,440  8  0-11  0-11  4.50  [4.34,  4.66]  4.00  3  3.11  9.67  .31  -.95  .81 | 1,446  2  0-24  0-24  5.63  [5.36,  5.90]  4.00  1  5.26  27.70  1.30  1.15  .90 | 1,439  8  0-24  0-24  4.75  [4.50,  5.00]  3.00  1  4.78  22.84  1.40  1.83  .89 | 1,409  39  0-50  0-49  16.97  [16.50,  17.43]  15.00  14  8.92  79.50  .66  .01  .74 | 1,400  48  0-50  0-47  16.93  [16.50,  17.35]  16.00  15  8.05  64.75  .50  .07  .72 | 1,446  2  0-30  0-30  3.71  [3.45,  3.97]  2.00  0  5.08  25.83  1.93  3.72  .89 | 1,433  15  0-30  0-30  3.72  [3.47,  3.96]  2.00  0  4.73  22.34  2.00  4.62  .89 |

*Note. N*, Number of individuals; PRNS, Parent-rated negative symptoms; One randomly selected twin per pair included in analyses; Data shown for sample included in phenotypic analyses who provided data at both time points.

**Table S3**. *Descriptives for psychotic experiences and negative symptoms subscales split by sex and zygosity*

|  | Paranoia | | | Hallucinations | | | Cognitive disorganization | | | Grandiosity | | | Anhedonia | | | PRNS | | | |
| --- | --- | --- | --- | --- | --- | --- | --- | --- | --- | --- | --- | --- | --- | --- | --- | --- | --- | --- | --- |
|  | *N* | *M* | *SD* | *N* | *M* | *SD* | *N* | *M* | *SD* | *N* | *M* | *SD* | *N* | *M* | *SD* | *N* | *M* | *SD* |  |
| Time 1  MZM  MZF  DZM  DZF  DZOS  Time 2  MZM  MZF  DZM  DZF  DZOS | 187  321  178  277  483  186  320  177  275  479 | 13.77  15.56  15.22  13.85  16.30  12.38  15.49  14.92  14.68  15.31 | 13.67  13.56  13.21  12.79  14.11  12.21  14.91  13.99  13.80  13.51 | 187  322  178  277  484  186  321  177  275  480 | 5.42  6.80  6.48  6.23  6.54  6.42  6.80  7.27  6.70  6.76 | 7.71  8.15  7.52  7.92  7.68  8.07  7.51  8.04  7.15  7.40 | 187  322  176  277  484  187  321  176  276  480 | 3.99  4.96  4.17  4.85  4.56  3.71  5.06  4.29  4.72  4.39 | 3.13  3.21  3.21  3.22  3.19  2.96  3.26  3.10  3.04  3.04 | 187  321  178  277  483  187  321  176  275  480 | 6.00  5.01  6.45  5.06  5.93  5.90  4.12  5.99  4.13  4.62 | 5.17  4.95  5.78  5.06  5.37  5.36  4.46  5.27  4.36  4.64 | 182  312  172  274  469  180  308  175  270  467 | 19.49  15.04  20.19  15.00  17.25  18.57  15.42  19.52  14.82  17.53 | 9.38  8.07  9.34  8.10  8.98  8.26  7.39  8.43  7.66  7.99 | 187  322  177  277  483  185  319  177  274  478 | 3.66  3.42  4.39  3.37  3.86  3.90  3.52  4.37  3.15  3.86 | 4.69  4.87  5.71  5.07  5.12  4.64  4.45  5.20  4.58  4.82 |  |

*Note. N,* Number of individuals; MZM, Monozygotic males; MZF, Monozygotic females; DZM, Dizygotic males; DZF, Dizygotic females; DZOS, Dizygotic opposite sex; PRNS, Parent-rated negative symptoms; One randomly selected twin per pair included in analyses; Data shown for sample included in phenotypic analyses who provided data at both time points.

**Table S4***.* *Frequencies of distress associated with psychotic experiences*

|  | *N* with PE score >0  and distress data | Not distressed | A bit distressed | Quite / very distressed |
| --- | --- | --- | --- | --- |
| Paranoia time 1  Paranoia time 2  Hallucinations time 1  Hallucinations time 2  Cognitive disorganization time 1  Cognitive disorganization time 2  Grandiosity time 1  Grandiosity time 2 | 1,026  1,335  776  1,127  1,001  1,283  979  1,221 | 666 (64.9%)  912 (68.3%)  586 (75.5%)  958 (85.0%)  627 (62.6%)  922 (71.9%)  837 (85.5%)  1,077 (88.2%) | 268 (26.1%)  320 (24.0%)  153 (19.7%)  145 (12.9%)  266 (26.6%)  289 (22.5%)  112 (11.4%)  105 (8.6%) | 92 (9.0%)  103 (7.7%)  37 (4.8%)  24 (2.1%)  108 (10.8%)  72 (5.6%)  30 (3.1%)  39 (3.2%) |

*Note. N,* Number of individuals; Distress data included only where psychotic experiences total subscale scores >0 at relative time-point; One randomly selected twin per pair included in analyses; Distress data not available for negative symptoms. PE, Psychotic experiences scale.

**Table S5***. Assumption testing for paranoia*

|  | | | | | Comparative fit with model in row above | | | | Comparative fit with saturated model | | | |
| --- | --- | --- | --- | --- | --- | --- | --- | --- | --- | --- | --- | --- |
| Model | -2LL | *df* | Parameters | BIC | ^Δ^χ*^2^* | ^Δ^*df* | *p* | AIC | ^Δ^χ*^2^* | ^Δ^*df* | *p* | AIC |
| Saturated  a  b  c  d  e | 43,244.21  43,261.15  43,266.15  43,282.94  43,323.36  43,335.36 | 16,398  16,406  16,414  16,422  16,430  16,434 | 70  62  54  46  38  34 | -100,473.86  -100,527.04  -100,592.15  -100,645.48  -100,675.17  -100,698.23 | -  16.94  5.01  16.78  40.43  11.99 | -  8  8  8  8  4 | -  .03  .76  .03  <.001  .02 | -  0.94  -10.99  0.78  24.43  3.99 | -  16.94  21.94  38.73  79.15  91.15 | -  8  16  24  32  36 | -  .03  .15  .03  <.001  <.001 | -  0.94  -10.06  -9.27  15.15  19.15 |

*Note.* Model definitions: a, equating means within twin pairs for same-sex pairs. b, equating variances within twin pairs for same-sex pairs. c, equating means and variances across zygosity for same-sex pairs (separating means and variances by sex). d, equating males in opposite-sex pairs with males in same-sex pairs (and the same for females). e, testing for sex differences in means and variances. −2LL, likelihood-ratio test (minus two times the log-likelihood of the data); BIC, Bayesian Information Criteria; AIC, Akaike’s Information Criteria*; df,* degrees of freedom; ^Δ^χ*^2^*, difference in −2LL statistic between models; ^Δ^*df*, difference in degrees of freedom between models; AIC calculated as ^Δ^χ*^2^* minus twice ^Δ^*df*.

**Table S6***. Assumption testing for hallucinations*

|  | | | | | Comparative fit with model in row above | | | | Comparative fit with saturated model | | | |
| --- | --- | --- | --- | --- | --- | --- | --- | --- | --- | --- | --- | --- |
| Model | -2LL | *df* | Parameters | BIC | ^Δ^χ*^2^* | ^Δ^*df* | *p* | AIC | ^Δ^χ*^2^* | ^Δ^*df* | *p* | AIC |
| Saturated  a  b  c  d  e | 43,756.14  43,770.46  43,773.82  43,780.97  43,821.65  43,841.01 | 16,413  16,421  16,429  16,437  16,445  16,449 | 70  62  54  46  38  34 | -100,093.40  -100,149.19  -100,215.95  -100,278.92  -100,308.34  -100,324.04 | -  14.32  3.36  7.15  40.69  19.36 | -  8  8  8  8  4 | -  .07  .91  .52  <.001  <.001 | -  -1.68  -12.64  -8.85  24.69  11.36 | -  14.32  17.68  24.83  65.52  84.88 | -  8  16  24  32  36 | -  .07  .34  .42  <.001  <.001 | -  -1.68  -14.32  -23.17  1.52  12.88 |

*Note.* Model definitions: a, equating means within twin pairs for same-sex pairs. b, equating variances within twin pairs for same-sex pairs. c, equating means and variances across zygosity for same-sex pairs (separating means and variances by sex). d, equating males in opposite-sex pairs with males in same-sex pairs (and the same for females). e, testing for sex differences in means and variances. −2LL, likelihood-ratio test (minus two times the log-likelihood of the data); BIC, Bayesian Information Criteria; AIC, Akaike’s Information Criteria*; df,* degrees of freedom; ^Δ^χ*^2^*, difference in −2LL statistic between models; ^Δ^*df*, difference in degrees of freedom between models; AIC calculated as ^Δ^χ*^2^* minus twice ^Δ^*df*.

**Table S7***. Assumption testing for cognitive disorganization*

|  | | | | | Comparative fit with model in row above | | | | Comparative fit with saturated model | | | |
| --- | --- | --- | --- | --- | --- | --- | --- | --- | --- | --- | --- | --- |
| Model | -2LL | *df* | Parameters | BIC | ^Δ^χ*^2^* | ^Δ^*df* | *p* | AIC | ^Δ^χ*^2^* | ^Δ^*df* | *p* | AIC |
| Saturated  a  b  c  d  e | 42,957.75  42,971.74  42,978.96  42,994.45  43,064.49  43,092.82 | 16,401  16,409  16,417  16,425  16,433  16,437 | 70  62  54  46  38  34 | -100,786.61  -100,842.73  -100,905.63  -100,960.26  -100,960.33  -100,967.06 | -  14.00  7.22  15.49  70.04  28.33 | -  8  8  8  8  4 | -  .08  .51  .05  <.001  <.001 | -  -2.00  -8.78  0.51  54.04  20.33 | -  14.00  21.21  38.70  106.74  135.07 | -  8  16  24  32  36 | -  .08  .17  .05  <.001  <.001 | -  -2.00  -10.79  -9.30  42.74  63.07 |

*Note.* Model definitions: a, equating means within twin pairs for same-sex pairs. b, equating variances within twin pairs for same-sex pairs. c, equating means and variances across zygosity for same-sex pairs (separating means and variances by sex). d, equating males in opposite-sex pairs with males in same-sex pairs (and the same for females). e, testing for sex differences in means and variances. −2LL, likelihood-ratio test (minus two times the log-likelihood of the data); BIC, Bayesian Information Criteria; AIC, Akaike’s Information Criteria*; df,* degrees of freedom; ^Δ^χ*^2^*, difference in −2LL statistic between models; ^Δ^*df*, difference in degrees of freedom between models; AIC calculated as ^Δ^χ*^2^* minus twice ^Δ^*df*.

**Table S8***. Assumption testing for grandiosity*

|  | | | | | Comparative fit with model in row above | | | | Comparative fit with saturated model | | | |
| --- | --- | --- | --- | --- | --- | --- | --- | --- | --- | --- | --- | --- |
| Model | -2LL | *df* | Parameters | BIC | ^Δ^χ*^2^* | ^Δ^*df* | *p* | AIC | ^Δ^χ*^2^* | ^Δ^*df* | *p* | AIC |
| Saturated  a  b  c  d  e | 43,969.74  43,973.67  43,978.71  43,989.83  43,998.76  44,001.92 | 16,402  16,410  16,418  16,426  16,434  16,438 | 70  62  54  46  38  34 | -99,783.39  -99,849.57  -99,914.64  -99,973.64  -100,034.82  -100,066.73 | -  3.93  5.04  11.12  8.93  3.16 | -  8  8  8  8  4 | -  .86  .75  .19  .34  .53 | -  -12.07  -10.96  -4.88  -7.07  -4.84 | -  3.93  8.97  20.09  29.02  32.18 | -  8  16  24  32  36 | -  .86  .92  .69  .62  .65 | -  -12.07  -23.03  -27.91  -34.98  -39.82 |

*Note.* Model definitions: a, equating means within twin pairs for same-sex pairs. b, equating variances within twin pairs for same-sex pairs. c, equating means and variances across zygosity for same-sex pairs (separating means and variances by sex). d, equating males in opposite-sex pairs with males in same-sex pairs (and the same for females). e, testing for sex differences in means and variances. −2LL, likelihood-ratio test (minus two times the log-likelihood of the data); BIC, Bayesian Information Criteria; AIC, Akaike’s Information Criteria*; df,* degrees of freedom; ^Δ^χ*^2^*, difference in −2LL statistic between models; ^Δ^*df*, difference in degrees of freedom between models; AIC calculated as ^Δ^χ*^2^* minus twice ^Δ^*df*.

**Table S9***. Assumption testing for anhedonia*

|  | | | | | Comparative fit with model in row above | | | | Comparative fit with saturated model | | | |
| --- | --- | --- | --- | --- | --- | --- | --- | --- | --- | --- | --- | --- |
| Model | -2LL | *df* | Parameters | BIC | ^Δ^χ*^2^* | ^Δ^*df* | *p* | AIC | ^Δ^χ*^2^* | ^Δ^*df* | *p* | AIC |
| Saturated  a  b  c  d  e | 43,667.51  43,668.60  43,681.07  43,684.75  43,696.87  43,699.35 | 16,410  16,418  16,426  16,434  16,442  16,446 | 70  62  54  46  38  34 | -100,155.74  -100,224.75  -100,282.40  -100,348.83  -100,406.83  -100,439.41 | -  1.10  12.47  3.68  12.12  2.48 | -  8  8  8  8  4 | -  .99  .13  .88  .15  .65 | -  -14.90  -3.53  -12.32  -3.88  -5.52 | -  1.10  13.57  17.25  29.37  31.85 | -  8  16  24  32  36 | -  .99  .63  .84  .60  .67 | -  -14.90  -18.43  -30.75  -34.63  -40.15 |

*Note.* Model definitions: a, equating means within twin pairs for same-sex pairs. b, equating variances within twin pairs for same-sex pairs. c, equating means and variances across zygosity for same-sex pairs (separating means and variances by sex). d, equating males in opposite-sex pairs with males in same-sex pairs (and the same for females). e, testing for sex differences in means and variances. −2LL, likelihood-ratio test (minus two times the log-likelihood of the data); BIC, Bayesian Information Criteria; AIC, Akaike’s Information Criteria*; df,* degrees of freedom; ^Δ^χ*^2^*, difference in −2LL statistic between models; ^Δ^*df*, difference in degrees of freedom between models; AIC calculated as ^Δ^χ*^2^* minus twice ^Δ^*df*.

**Table S10***. Assumption testing for parent-rated negative symptoms*

|  | | | | | Comparative fit with model in row above | | | | Comparative fit with saturated model | | | |
| --- | --- | --- | --- | --- | --- | --- | --- | --- | --- | --- | --- | --- |
| Model | -2LL | *df* | Parameters | BIC | ^Δ^χ*^2^* | ^Δ^*df* | *p* | AIC | ^Δ^χ*^2^* | ^Δ^*df* | *p* | AIC |
| Saturated  a  b  c  d  e | 40,075.14  40,122.03  40,131.52  40,140.88  40,178.75  40,205.28 | 16,454  16,462  16,470  16,478  16,486  16,490 | 70  62  54  46  38  34 | -104,133.73  -104,156.96  -104,217.59  -104,278.34  -104,310.59  -104,319.11 | -  46.89  9.49  9.37  37.86  26.53 | -  8  8  8  8  4 | -  <.001  .30  .31  <.001  <.001 | -  30.89  -6.51  -6.63  21.86  18.53 | -  46.89  56.38  65.74  103.60  130.14 | -  8  16  24  32  36 | -  <.001  <.001  <.001  <.001  <.001 | -  30.89  24.38  17.74  39.60  58.14 |

*Note.* Model definitions: a, equating means within twin pairs for same-sex pairs. b, equating variances within twin pairs for same-sex pairs. c, equating means and variances across zygosity for same-sex pairs (separating means and variances by sex). d, equating males in opposite-sex pairs with males in same-sex pairs (and the same for females). e, testing for sex differences in means and variances. −2LL, likelihood-ratio test (minus two times the log-likelihood of the data); BIC, Bayesian Information Criteria; AIC, Akaike’s Information Criteria*; df,* degrees of freedom; ^Δ^χ*^2^*, difference in −2LL statistic between models; ^Δ^*df*, difference in degrees of freedom between models; AIC calculated as ^Δ^χ*^2^* minus twice ^Δ^*df*.

**Table S11***. Twin model statistics for paranoia*

| Model | -2LL | *df* | Parameters | BIC | Comparative model | ^Δ^χ*^2^* | ^Δ^*df* | *p* | AIC |
| --- | --- | --- | --- | --- | --- | --- | --- | --- | --- |
| Saturated  Full ACE  Full ADE  ACE Quan  ACE Scalar  ACE Hom**  Submodels  AE  CE  E | 43,244.21  43,329.40  43,340.58  43,348.80  43,358.18  43,364.40  43,442.04  43,467.17  44,303.823 | 16,398  16,443  16,443  16,446  16,449  16,455  16,458  16,458  16,461 | 70  25  25  22  19  13  10  10  7 | -100,473.86  -100,783.06  -100,771.88  -100,789.96  -100,806.87  -100,853.24  -100,801.89  -100,776.77  -99,967.00 | -  Saturated  Saturated  Full ACE  ACE Quant  ACE Scalar  ACE Hom  ACE Hom  ACE Hom | -  85.19  96.37  19.40  9.38  6.22  77.65  102.77  938.83 | -  45  45  3  3  6  3  3  6 | -  <.001  <.001  <.001  .03  .40  <.001  <.001  <.001 | -  -4.81  6.37  13.40  3.38  -5.78  71.65  96.77  926.83 |

*Note.* Full ACE, ACE model with quantitative and qualitative sex differences; Full ADE, ADE model with quantitative and qualitative sex differences; ACE Quan, ACE model with quantitative sex differences; ACE Scalar, ACE model without sex differences on the aetiological correlations; ACE Hom, ACE model without sex differences; −2LL, likelihood-ratio test (minus two times the log-likelihood of the data); BIC, Bayesian Information Criteria; AIC, Akaike’s Information Criteria*; df,* degrees of freedom; ^Δ^χ*^2^*, difference in −2LL statistic between models; ^Δ^*df*, difference in degrees of freedom between models; AIC calculated as ^Δ^χ*^2^* minus twice ^Δ^*df*; **, best-fitting overall model; Full sex differences models are compared to the saturated model. Subsequent models are compared to and nested within the antecedent model. Submodels are compared to and nested within the best-fitting ACE / ADE model.

**Table S12***. Twin model statistics for hallucinations*

| Model | -2LL | *df* | Parameters | BIC | Comparative model | ^Δ^χ*^2^* | ^Δ^*df* | *p* | AIC |
| --- | --- | --- | --- | --- | --- | --- | --- | --- | --- |
| Saturated  Full ACE  ACE Quan  ACE Scalar  ACE Hom**  Submodels  AE  CE  E | 43,756.14  43,819.93  43,827.65  43,837.65  43,846.91  43,933.49  43,923.37  44,641.98 | 16,413  16,458  16,461  16,464  16,470  16,473  16,473  16,476 | 70  25  22  19  13  10  10  7 | -100,093.40  -100,424.00  -100,442.57  -100,458.87  -100,502.19  -100,441.91  -100,452.03  -99,759.71 | -  Saturated  Full ACE  ACE Quant  ACE Scalar  ACE Hom  ACE Hom  ACE Hom | -  63.79  7.72  10.00  9.26  86.58  76.46  795.07 | -  45  3  3  6  3  3  6 | -  .03  .05  .02  .16  <.001  <.001  <.001 | -  -26.21  1.72  4.00  -2.73  80.58  70.46  782.07 |

*Note.* Full ACE, ACE model with quantitative and qualitative sex differences; ACE Quan, ACE model with quantitative sex differences; ACE Scalar, ACE model without sex differences on the aetiological correlations; ACE Hom, ACE model without sex differences; −2LL, likelihood-ratio test (minus two times the log-likelihood of the data); BIC, Bayesian Information Criteria; AIC, Akaike’s Information Criteria*; df,* degrees of freedom; ^Δ^χ*^2^*, difference in −2LL statistic between models; ^Δ^*df*, difference in degrees of freedom between models; AIC calculated as ^Δ^χ*^2^* minus twice ^Δ^*df*; **, best-fitting overall model; Full sex differences models are compared to the saturated model. Subsequent models are compared to and nested within the antecedent model. Submodels are compared to and nested within the best-fitting ACE model.

**Table S13***. Twin model statistics for cognitive disorganization*

| Model | -2LL | *df* | Parameters | BIC | Comparative model | ^Δ^χ*^2^* | ^Δ^*df* | *p* | AIC |
| --- | --- | --- | --- | --- | --- | --- | --- | --- | --- |
| Saturated  Full ACE  Full ADE  ACE Quan  ACE Scalar  ACE Hom**  Submodels  AE  CE  E | 42,957.75  43,037.59  43,041.44  43,052.23  43,059.55  43,066.06  43,117.87  43,166.67  43,743.21 | 16,401  16,446  16,446  16,449  16,452  16,458  16,461  16,461  16,464 | 70  25  25  22  19  13  10  10  7 | -100,786.61  -101,101.17  -101,097.30  -101,112.83  -101,131.80  -101,177.87  -101,152.79  -101,103.56  -100,553.31 | -  Saturated  Saturated  Full ACE  ACE Quant  ACE Scalar  ACE Hom  ACE Hom  ACE Hom | -  79.84  83.69  14.64  7.32  6.51  51.81  100.61  677.15 | -  45  45  3  3  6  3  3  6 | -  <.001  <.00  <.001  .06  .37  <.001  <.001  <.001 | -  -10.16  -6.31  8.64  1.32  -5.78  45.38  94.61  665.15 |

*Note.* Full ACE, ACE model with quantitative and qualitative sex differences; Full ADE, ADE model with quantitative and qualitative sex differences; ACE Quan, ACE model with quantitative sex differences; ACE Scalar, ACE model without sex differences on the aetiological correlations; ACE Hom, ACE model without sex differences; −2LL, likelihood-ratio test (minus two times the log-likelihood of the data); BIC, Bayesian Information Criteria; AIC, Akaike’s Information Criteria*; df,* degrees of freedom; ^Δ^χ*^2^*, difference in −2LL statistic between models; ^Δ^*df*, difference in degrees of freedom between models; AIC calculated as ^Δ^χ*^2^* minus twice ^Δ^*df*; **, best-fitting overall model; Full sex differences models are compared to the saturated model. Subsequent models are compared to and nested within the antecedent model. Submodels are compared to and nested within the best-fitting ACE / ADE model.

**Table S14***. Twin model statistics for grandiosity*

| Model | -2LL | *df* | Parameters | BIC | Comparative model | ^Δ^χ*^2^* | ^Δ^*df* | *p* | AIC |
| --- | --- | --- | --- | --- | --- | --- | --- | --- | --- |
| Saturated  Full ACE  Full ADE  ACE Quan  ACE Scalar  ACE Hom**  Submodels  AE  CE  E | 43,969.74  44,017.11  44,044.02  44,036.19  43,041.75  44,057.15  44,140.66  44,141.66  44,887.09 | 16,402  16,447  16,447  16,450  16,453  16,459  16,462  16,462  16,465 | 70  25  25  22  19  13  10  10  7 | -99,783.39  -100,130.41  -100,103.50  -100,137.63  -100,158.36  -100,195.55  -100,138.33  -100,137.33  -99,418.19 | -  Saturated  Saturated  Full ACE  ACE Quant  ACE Scalar  ACE Hom  ACE Hom  ACE Hom | -  47.37  74.28  19.08  5.56  15.40  83.51  84.51  829.94 | -  45  45  3  3  6  3  3  6 | -  .38  <.001  <.001  .14  .02  <.001  <.001  <.001 | -  -42.63  -15.72  13.08  -0.44  3.40  77.51  78.51  817.94 |

*Note.* Full ACE, ACE model with quantitative and qualitative sex differences; Full ADE, ADE model with quantitative and qualitative sex differences; ACE Quan, ACE model with quantitative sex differences; ACE Scalar, ACE model without sex differences on the aetiological correlations; ACE Hom, ACE model without sex differences; −2LL, likelihood-ratio test (minus two times the log-likelihood of the data); BIC, Bayesian Information Criteria; AIC, Akaike’s Information Criteria*; df,* degrees of freedom; ^Δ^χ*^2^*, difference in −2LL statistic between models; ^Δ^*df*, difference in degrees of freedom between models; AIC calculated as ^Δ^χ*^2^* minus twice ^Δ^*df*; **, best-fitting overall model; Full sex differences models are compared to the saturated model. Subsequent models are compared to and nested within the antecedent model. Submodels are compared to and nested within the best-fitting ACE / ADE model.

**Table S15***. Twin model statistics for anhedonia*

| Model | -2LL | *df* | Parameters | BIC | Comparative model | ^Δ^χ*^2^* | ^Δ^*df* | *p* | AIC |
| --- | --- | --- | --- | --- | --- | --- | --- | --- | --- |
| Saturated  Full ACE  Full ADE  ADE Quan  ADE Scalar  ADE Hom  Submodels  AE**  E | 43,667.51  43,741.63  44,723.41  43,735.51  43,743.33  43,750.27  43,785.25  44,457.19 | 16,410  16,455  16,455  16,458  16,461  16,467  16,470  16,473 | 70  25  25  22  19  13  10  7 | -100,155.74  -100,476.00  -100,494.23  -100,508.42  -100,526.89  -100,572.54  -100,563.86  -99,918.21 | -  Saturated  Saturated  Full ADE  ADE Quant  ADE Scalar  ADE Hom  ADE Hom | -  74.12  55.91  12.10  7.82  6.94  34.98  706.92 | -  45  45  3  3  6  3  6 | -  <.001  .13  .01  .05  .33  <.001  <.001 | -  -15.88  -34.09  6.10  1.82  -5.06  28.98  694.92 |

*Note.* Full ACE, ACE model with quantitative and qualitative sex differences; Full ADE, ADE model with quantitative and qualitative sex differences; ACE Quan, ACE model with quantitative sex differences; ACE Scalar, ACE model without sex differences on the aetiological correlations; ACE Hom, ACE model without sex differences; −2LL, likelihood-ratio test (minus two times the log-likelihood of the data); BIC, Bayesian Information Criteria; AIC, Akaike’s Information Criteria*; df,* degrees of freedom; ^Δ^χ*^2^*, difference in −2LL statistic between models; ^Δ^*df*, difference in degrees of freedom between models; AIC calculated as ^Δ^χ*^2^* minus twice ^Δ^*df*; **, best-fitting overall model; Full sex differences models are compared to the saturated model. Subsequent models are compared to and nested within the antecedent model. Submodels are compared to and nested within the best-fitting ACE / ADE model.

**Table S16***. Twin model statistics for parent-rated negative symptoms*

| Model | -2LL | *df* | Parameters | BIC | Comparative model | ^Δ^χ*^2^* | ^Δ^*df* | *p* | AIC |
| --- | --- | --- | --- | --- | --- | --- | --- | --- | --- |
| Saturated  Full ACE  ACE Quan  ACE Scalar  ACE Hom**  Submodels  AE  CE  E | 40,075.14  40,187.65  40,268.73  40,268.85  40,283.92  40,867.24  40,887.89  44,531.44 | 16,454  16,499  16,502  16,505  16,511  16,514  16,514  16,517 | 70  25  22  19  13  10  10  7 | -104,133.73  -104,415.62  -104,360.83  -104,387.01  -104,424.53  -103,867.49  -103,846.85  -100,229.59 | -  Saturated  Full ACE  ACE Quant  ACE Scalar  ACE Hom  ACE Hom  ACE Hom | -  112.51  81.08  0.12  15.07  583.32  603.97  4,247.52 | -  45  3  3  6  3  3  6 | -  <.001  <.001  .99  .02  <.001  <.001  <.001 | -  22.51  75.08  -5.88  3.07  577.32  597.97  4235.52 |

*Note.* Full ACE, ACE model with quantitative and qualitative sex differences; ACE Quan, ACE model with quantitative sex differences; ACE Scalar, ACE model without sex differences on the aetiological correlations; ACE Hom, ACE model without sex differences; −2LL, likelihood-ratio test (minus two times the log-likelihood of the data); BIC, Bayesian Information Criteria; AIC, Akaike’s Information Criteria*; df,* degrees of freedom; ^Δ^χ*^2^*, difference in −2LL statistic between models; ^Δ^*df*, difference in degrees of freedom between models; AIC calculated as ^Δ^χ*^2^* minus twice ^Δ^*df*; **, best-fitting overall model; Full sex differences models are compared to the saturated model. Subsequent models are compared to and nested within the antecedent model. Submodels are compared to and nested within the best-fitting ACE model.

**Table S17***.* *Cholesky estimates*

|  | Total shared | Total unique | Shared A | Unique A | Shared C | Unique C | Shared E | Unique E |
| --- | --- | --- | --- | --- | --- | --- | --- | --- |
| Paranoia  Hallucinations  Cognitive disorganization  Grandiosity  Anhedonia  PRNS | .44  .46  .58  .44  .42  .43 | .56  .54  .43  .57  .58  .57 | .25  .25  .38  .26  .32  .27 | .08  .08  .00  .00  .13  .18 | .08  .12  .06  .11  -  .13 | .04  .04  .00  .08  -  .25 | .11  .09  .14  .07  .10  .03 | .44  .42  .43  .49  .44  .14 |

*Note.* A, Additive genetic effects; C, Common environmental effects; E, Nonshared environmental effects; PRNS, Parent-rated negative symptoms; Cholesky estimates represent the proportion of variation at time 2 that is due to influences shared with time 1, and those that are unique to time 2.

**Table S18***.* *Genetic and environmental correlations for best-fitting bivariate models*

|  | *r*A | *r*C | *r*E |
| --- | --- | --- | --- |
| Paranoia  Hallucinations  Cognitive disorganization  Grandiosity  Anhedonia  PRNS | .87 [.73, 1.00]  .87 [.70, 1.00]  1.00 [.92, 1.00]  1.00 [.84, 1.00]  .84 [.79, .90]  .77 [.72, .83] | .83 [.64, 1.00]  .87 [.71, 1.00]  1.00 [.84, 1.00]  .76 [.63, .91]  -  .59 [.52, .65] | .45 [.39, .50]  .41 [.35, .46]  .49 [.45, .53]  .36 [.31, .41]  .44 [.39, .49]  .45 [.38, .51] |

*Note*. *r*A, Genetic correlation; *r*C, Common environment correlation; *r*E, Nonshared environment correlation; PRNS, Parent-rated negative symptoms; 95% CI in parentheses.

**Table S19***.* *Descriptives and mean differences for psychotic experiences and negative symptoms subscales at time 1 by group*

|  | Group | | | |
| --- | --- | --- | --- | --- |
|  | Low-scoring (LS) | Increasing (I) | Decreasing (D) | Persistent (P) |
| Paranoia  *N* (%)  Paranoia mean score (*SD*)  Cohen’s *d* [95% CI]  Hallucinations  *N* (%)  Hallucinations mean score (*SD*)  Cohen’s *d* [95% CI]  Cognitive disorganization  *N* (%)  Cognitive disorganization mean score (*SD*)  Cohen’s *d* [95% CI]  Grandiosity  *N* (%)  Grandiosity mean score (*SD*)  Cohen’s *d* [95% CI]  Anhedonia  *N* (%)  Anhedonia mean score (*SD*)  Cohen’s *d* [95% CI]  PRNS  *N* (%)  PRNS mean score (*SD*)  Cohen’s *d* [95% CI] | 1,215 (84.7%)  11.09 (8.85)  LS versus I = 1.25 [0.99, 1.50]  1,182 (82.1%)  3.61 (4.21)  LS versus I = 1.61 [1.39, 1.84]  1,138 (79.1%)  3.42 (2.37)  LS versus I = 1.30 [1.06, 1.53]  1,212 (84.3%)  3.96 (3.14)  LS versus I = 1.08 [0.82, 1.34]  1,141 (83.5%)  14.25 (6.39)  LS versus I = 1.23 [1.00, 1.46]  1,173 (81.9%)  1.91 (2.35)  LS versus I = 1.48 [1.27, 1.70]) | 64 (4.5%)  22.19 (9.71)  86 (6.0%)  10.49 (4.99)  79 (5.5%)  6.45 (1.74)  61 (4.3%)  7.36 (3.30)  81 (5.9%)  22.05 (5.66)  97 (6.8%)  5.48 (3.03) | 77 (5.4%)  42.87 (5.89)  D versus P = 0.41 [0.09, 0.72]  92 (6.4%)  22.57 (5.15)  D versus P = 0.31 [0.01, 0.61]  105 (7.3%)  9.67 (0.70)  D versus P = 0.56 [0.29, 0.82]  72 (5.0%)  16.81 (2.93)  D versus P = 0.27 [-0.04, 0.58]  63 (4.6%)  34.22 (2.83)  D versus P = 0.50 [0.17, 0.84]  66 (4.6%)  14.69 (3.55)  D versus P = 0.37 [0.05, 0.68] | 79 (5.5%)  45.76 (8.17)  79 (5.5%)  24.23 (5.68)  116 (8.1%)  10.07 (0.74)  92 (6.4%)  17.57 (2.81)  82 (6.0%)  35.74 (3.17)  96 (6.7%)  16.10 (4.05) |

*Note. N,* Number of individuals; PRNS, Parent-rated negative symptoms; LS, Low-scoring group; I, Increasing group; D, Decreasing group; P, Persistent group. Cohen’s *d* measure of strength of association for unequal sample sizes (effect size of the standardized difference between the means; equal to the difference between the means divided by pooled *SD*); One randomly selected twin per pair included in analyses.

**Table S20***.* *Mean differences for depression traits at time 1 by group*

|  | Group | | | |
| --- | --- | --- | --- | --- |
|  | Low-scoring (LS) | Increasing (I) | Decreasing (D) | Persistent (P) |
| Paranoia  *N* (%)  SMFQ mean score (*SD*)  Cohen’s *d* [95% CI]  Hallucinations  *N* (%)  SMFQ mean score (*SD*)  Cohen’s *d* [95% CI]  Cognitive disorganization  *N* (%)  SMFQ mean score (*SD*)  Cohen’s *d* [95% CI]  Grandiosity  *N* (%)  SMFQ mean score (*SD*)  Cohen’s *d* [95% CI]  Anhedonia  *N* (%)  SMFQ mean score (*SD*)  Cohen’s *d* [95% CI]  PRNS  *N* (%)  SMFQ mean score (*SD*)  Cohen’s *d* [95% CI] | 1,214 (84.7%)  3.89 (4.67)  LS versus I = 0.84 [0.59, 1.10])  1,181 (82.1%)  4.03 (4.95)  LS versus I = 0.80 [0.58, 1.02]  1,137 (79.1%)  3.53 (4.34)  LS versus I = 0.95 [0.72, 1.18]  1,211 (84.3%)  4.86 (5.51)  LS versus I = -0.02 [-0.27, 0.24]  1,140 (83.5%)  4.53 (5.22)  LS versus I = 0.13 [-0.09, 0.36]  1,172 (81.9%)  4.46 (5.35)  LS versus I = 0.38 [0.17, 0.59] | 64 (4.5%)  7.92 (6.44)  I versus P = 0.59 [0.26, 0.93]  86 (6.0%)  8.11 (6.69)  I versus P = 0.24 [-0.07, 0.54]  79 (5.5%)  7.83 (6.56)  I versus P = 0.51 [0.22, 0.80]  61 (4.2%)  4.78 (5.43)  I versus P = 0.06 [-0.39, 0.23]  81 (5.9%)  5.22 (5.17)  I versus P = 0.30 [-0.01, 0.61]  97 (6.8%)  6.49 (5.70)  I versus P = 0.08 [-0.20, 0.37] | 77 (5.4%)  9.88 (5.49)  D versus P = 0.33 [0.02, 0.65]  92 (6.4%)  7.89 (5.41)  D versus P = 0.30 [-0.01, 0.60]  105 (7.3%)  9.68 (5.84)  D versus P = 0.24 [-0.02, 0.51]  72 (5.0%)  4.97 (5.45)  D versus P = 0.10 [-0.41, 0.21]  63 (4.6%)  6.39 (6.78)  D versus P = 0.10 [-0.23, 0.43]  66 (4.6%)  5.86 (5.23)  D versus P = 0.20 [-0.12, 0.51] | 79 (5.5%)  12.01 (7.24)  LS versus P = 1.67 [1.43, 1.91]  79 (5.5%)  9.69 (6.65)  LS versus P = 1.12 [0.88, 1.35]  116 (8.1%)  11.21 (6.62)  LS versus P = 1.67 [1.47, 1.87]  92 (6.4%)  4.41 (5.38)  LS versus P = -0.08 [-0.29, 0.13]  82 (6.0%)  7.05 (6.82)  LS versus P = 0.47 [0.25, 0.70]  96 (6.7%)  6.99 (6.13)  LS versus P = 0.47 [0.26, 0.68] |

*Note. N*, Number of individuals; PRNS, Parent-rated negative symptoms; SMFQ, Short Mood and Feeling Questionnaire (self-rated) measure of depression traits; LS, Low-scoring group; I, Increasing group; D, Decreasing group; P, Persistent group. Cohen’s *d* measure of strength of association for unequal sample sizes (effect size of the standardized difference between the means; equal to the difference between the means divided by pooled *SD*); One randomly selected twin per pair included in analyses; SMFQ score at time 1 for whole sample: Mean = 4.83; *SD* = 5.49 (*N* = 1,447).

**Table S21***. Mean differences for emotional problems at time 1 by group*

|  | Group | | | |
| --- | --- | --- | --- | --- |
|  | Low-scoring (LS) | Increasing (I) | Decreasing (D) | Persistent (P) |
| Paranoia  *N* (%)  SDQ mean score (*SD*)  Cohen’s *d* [95% CI]  Hallucinations  *N* (%)  SDQ mean score (*SD*)  Cohen’s *d* [95% CI]  Cognitive disorganization  *N* (%)  SDQ mean score (*SD*)  Cohen’s *d* [95% CI]  Grandiosity  *N* (%)  SDQ mean score (*SD*)  Cohen’s *d* [95% CI]  Anhedonia  *N* (%)  SDQ mean score (*SD*)  Cohen’s *d* [95% CI]  PRNS  *N* (%)  SDQ mean score (*SD*)  Cohen’s *d* [95% CI] | 1,213 (84.8%)  2.81 (2.26)  LS versus I = 0.62 [0.36, 0.87]  1,179 (82.2%)  2.85 (2.30)  LS versus I = 0.61 [0.39, 0.83]  1,13*5* (79.1%)  2.61 (2.19)  LS versus I = 0.74 [0.51, 0.97]  1,209 (84.4%)  3.20 (2.45)  LS versus I = -0.05 [-0.31, 0.21]  1,139 (83.5%)  3.11 (2.42)  LS versus I = 0.06 [-0.17, 0.28]  1,170 (81.9%)  3.01 (2.44)  LS versus I = 0.19 [-0.02, 0.40] | 63 (4.4%)  4.22 (2.61)  I versus P = 0.63 [0.29, 0.97]  86 (6.0%)  4.28 (2.81)  I versus P = 0.38 [0.07, 0.69]  79 (5.5%)  4.23 (2.14)  I versus P = 0.74 [0.45, 1.04]  60 (4.2%)  3.08 (2.23)  I versus P = 0.19 [-0.51, 0.14]  81 (5.9%)  3.25 (2.48)  I versus P = 0.07 [-0.23, 0.39]  97 (6.8%)  3.47 (2.28)  I versus P = 0.29 [0.01, 0.58] | 77 (5.4%)  4.95 (2.47)  D versus P = 0.36 [0.04, 0.67]  92 (6.4%)  4.16 (2.36)  D versus P = 0.46 [0.16, 0.77]  105 (7.3%)  5.31 (2.32)  D versus P = 0.21 [-0.06, 0.47]  72 (5.0%)  3.03 (2.52)  D versus P = -0.16 [-0.47, 0.15]  62 (4.6%)  3.59 (2.70)  D versus P = 0.05 [-0.38, 0.28]  65 (4.6%)  3.72 (2.30)  D versus P = 0.19 [-0.13, 0.50] | 78 (5.5%)  5.85 (2.59)  LS versus P = 1.33 [1.10, 1.57]  78 (5.4%)  5.31 (2.61)  LS versus P = 1.06 [0.83, 1.29]  115 (8.0%)  5.82 (2.14)  LS versus P = 1.47 [1.27, 1.67]  92 (6.4%)  2.64 (2.48)  LS versus P = -0.23 [-0.44, -0.02]  82 (6.0%)  3.45 (2.62)  LS versus P = 0.14 [-0.09, 0.36]  96 (6.7%)  4.17 (2.52)  LS versus P = 0.47 [0.27, 0.68] |

*Note. N,* Number of individuals; PRNS, Parent-rated negative symptoms; SDQ emotional problems, Strengths and Difficulty Questionnaire emotional problems subscale (self-rated); LS, Low-scoring group; I, Increasing group; D, Decreasing group; P, Persistent group. Cohen’s *d* measure of strength of association for unequal sample sizes (effect size of the standardized difference between the means; equal to the difference between the means divided by pooled *SD*); One randomly selected twin per pair included in analyses; SDQ emotional problems score at time 1 for whole sample: Mean = 3.16; *SD* = 2.45 (*N =* 1,444).

**Table S22**. *Mean differences for conduct problems at time 1 by group*

|  | Group | | | |
| --- | --- | --- | --- | --- |
|  | Low-scoring (LS) | Increasing (I) | Decreasing (D) | Persistent (P) |
| Paranoia  *N* (%)  SDQ mean score (*SD*)  Cohen’s *d* [95% CI]  Hallucinations  *N* (%)  SDQ mean score (*SD*)  Cohen’s *d* [95% CI]  Cognitive disorganization  *N* (%)  SDQ mean score (*SD*)  Cohen’s *d* [95% CI]  Grandiosity  *N* (%)  SDQ mean score (*SD*)  Cohen’s *d* [95% CI]  Anhedonia  *N* (%)  SDQ mean score (*SD*)  Cohen’s *d* [95% CI]  PRNS  *N* (%)  SDQ mean score (*SD*)  Cohen’s *d* [95% CI] | 1,213 (84.7%)  1.63 (1.48)  LS versus I = 0.76 [0.65, 0.88])  1,179 (82.1%)  1.61 (1.44)  LS versus I = 0.85 [0.73, 0.96]  1,135 (79.1%)  1.58 (1.44)  LS versus I = 0.44 [0.33, 0.55]  1,209 (84.3%)  1.76 (1.55)  LS versus I = 0.28 [0.17 ,0.39]  1,139 (83.5%)  1.77 (1.55)  LS versus I = 0.17 [0.06, 0.28]  1,170 (81.9%)  1.71 (1.54)  LS versus I = 0.22 [0.11, 0.33] | 63 (4.5%)  2.94 (1.92)  I versus P = 0.13 [-0.20, 0.46]  86 (6.0%)  3.01 (1.84)  I versus P = -0.16 [-0.46, 0.15]  79 (5.5%)  2.25 (1.60)  I versus P = 0.43 [0.14, 0.71]  60 (4.2%)  2.22 (1.76)  I versus P = -0.01 [-0.33, 0.31]  81 (5.9%)  2.06 (1.84)  I versus P = 0.17 [-0.14, 0.48]  97 (6.8%)  2.06 (1.59)  I versus P = 0.34 [0.06, 0.63] | 77 (5.4%)  2.70 (1.71)  D versus P = 0.28 [-0.04, 0.59]  92 (6.4%)  2.79 (1.88)  D versus P = -0.04 [-0.34, 0.26]  105 (7.3%)  3.02 (1.60)  D versus P = -0.04 [-0.31, 0.22]  72 (5.0%)  2.35 (2.06)  D versus P = -0.08 [-0.39, 0.23]  62 (4.6%)  2.03 (1.59)  D versus P = 0.20 [-0.13, 0.53]  65 (4.6%)  2.53 (1.94)  D versus P = 0.06 [-0.25, 0.37] | 78 (5.5%)  3.18 (1.78)  LS versus P = 0.95 [0.83, 1.06]  78 (5.5%)  2.71 (2.02)  LS versus P = 0.63 [0.51, 0.74]  115 (8.1%)  2.95 (1.68)  LS versus P = 0.88 [0.76, 0.99]  92 (6.4%)  2.20 (1.65)  LS versus P = 0.28 [0.17, 0.38]  82 (6.0%)  2.38 (1.90)  LS versus P = 0.35 [0.24, 0.47]  96 (6.7%)  2.65 (1.84)  LS versus P = 0.55 [0.44, 0.68] |

*Note. N,* Number of individuals; PRNS, Parent-rated negative symptoms; SDQ conduct problems, Strengths and Difficulty Questionnaire conduct problems subscale (self-rated); LS, Low-scoring group; I, Increasing group; D, Decreasing group; P, Persistent group. Cohen’s *d* measure of strength of association for unequal sample sizes (effect size of the standardized difference between the means; equal to the difference between the means divided by pooled *SD*); One randomly selected twin per pair included in analyses.

**Table S23**. *Mean differences for hyperactivity at time 1 by group*

|  | Group | | | |
| --- | --- | --- | --- | --- |
|  | Low-scoring (LS) | Increasing (I) | Decreasing (D) | Persistent (P) |
| Paranoia  *N* (%)  SDQ mean score (*SD*)  Cohen’s *d* [95% CI]  Hallucinations  *N* (%)  SDQ mean score (*SD*)  Cohen’s *d* [95% CI]  Cognitive disorganization  *N* (%)  SDQ mean score (*SD*)  Cohen’s *d* [95% CI]  Grandiosity  *N* (%)  SDQ mean score (*SD*)  Cohen’s *d* [95% CI]  Anhedonia  *N* (%)  SDQ mean score (*SD*)  Cohen’s *d* [95% CI]  PRNS  *N* (%)  SDQ mean score (*SD*)  Cohen’s *d* [95% CI] | 1,213 (84.7%)  3.62 (2.45)  LS versus I = 0.60 [0.49, 0.71])  1,179 (82.1%)  3.59 (2.43)  LS versus I = 0.58 [0.47, 0.70]  1,135 (79.1%)  3.31 (2.26)  LS versus I = 0.81 [0.69, 0.92]  1,209 (84.3%)  3.82 (2.46)  LS versus I = 0.06 [-0.05 ,0.17]  1,139 (83.5%)  3.77 (2.47)  LS versus I = 0.39 [0.28, 0.50]  1,170 (81.9%)  3.69 (2.50)  LS versus I = 0.29 [0.18, 0.40] | 63 (4.5%)  5.10 (2.51)  I versus P = 0.15 [-0.16, 0.45]  86 (6.0%)  5.01 (2.45)  I versus P = 0.06 [-0.24, 0.37]  79 (5.5%)  5.13 (2.24)  I versus P = 0.67 [0.38, 0.96]  60 (4.2%)  3.96 (2.35)  I versus P = -0.02 [-0.34, 0.30]  81 (5.9%)  4.76 (2.60)  I versus P = -0.26 [-0.57, 0.]  97 (6.8%)  4.42 (2.52)  I versus P = 0.16 [-0.12, 0.45] | 77 (5.4%)  5.11 (2.30)  D versus P = 0.15 [-0.17, 0.47]  92 (6.4%)  5.17 (2.39)  D versus P = 0.00 [-0.30, 0.30]  105 (7.3%)  5.97 (2.22)  D versus P = 0.29 [0.02, 0.55]  72 (5.0%)  4.47 (2.90)  D versus P = -0.20 [-0.51, 0.11]  62 (4.6%)  3.84 (2.67)  D versus P = 0.11 [-0.22, 0.43]  65 (4.6%)  4.74 (2.24)  D versus P = 0.04 [-0.27, 0.35] | 78 (5.5%)  5.46 (2.39)  LS versus P = 0.76 [0.65, 0.87]  78 (5.5%)  5.17 (2.70)  LS versus P = 0.62 [0.50, 0.73]  115 (8.1%)  6.59 (2.12)  LS versus P = 1.50 [1.37, 1.62]  92 (6.4%)  3.90 (2.81)  LS versus P = 0.03 [-0.08, 0.14]  82 (6.0%)  4.11 (2.42)  LS versus P = 0.14 [0.03, 0.25]  96 (6.7%)  4.83 (2.51)  LS versus P = 0.46 [0.34, 0.57] |

*Note. N,* Number of individuals; PRNS, Parent-rated negative symptoms; SDQ hyperactivity, Strengths and Difficulty Questionnaire hyperactivity subscale (self-rated); LS, Low-scoring group; I, Increasing group; D, Decreasing group; P, Persistent group. Cohen’s *d* measure of strength of association for unequal sample sizes (effect size of the standardized difference between the means; equal to the difference between the means divided by pooled *SD*); One randomly selected twin per pair included in analyses.

**Table S24** *Mean differences for peer problems at time 1 by group*

|  | Group | | | |
| --- | --- | --- | --- | --- |
|  | Low-scoring (LS) | Increasing (I) | Decreasing (D) | Persistent (P) |
| Paranoia  *N* (%)  SDQ mean score (*SD*)  Cohen’s *d* [95% CI]  Hallucinations  *N* (%)  SDQ mean score (*SD*)  Cohen’s *d* [95% CI]  Cognitive disorganization  *N* (%)  SDQ mean score (*SD*)  Cohen’s *d* [95% CI]  Grandiosity  *N* (%)  SDQ mean score (*SD*)  Cohen’s *d* [95% CI]  Anhedonia  *N* (%)  SDQ mean score (*SD*)  Cohen’s *d* [95% CI]  PRNS  *N* (%)  SDQ mean score (*SD*)  Cohen’s *d* [95% CI] | 1,214 (84.7%)  1.60 (1.54)  LS versus I = 0.52 [0.44, 0.60])  1,179 (82.1%)  1.73 (1.73)  LS versus I = 0.28 [0.17, 0.39]  1,135 (79.1%)  1.63 (1.64)  LS versus I = 0.43 [0.32, 0.55]  1,209 (84.3%)  1.84 (1.73)  LS versus I = 0.11 [-0.01 ,0.22]  1,139 (83.5%)  1.74 (1.72)  LS versus I = 0.31 [0.19, 0.42]  1,170 (81.9%)  1.70 (1.72)  LS versus I = 0.57 [0.45, 0.68] | 63 (4.5%)  2.52 (1.97)  I versus P = 0.69 [0.35-1.02]  86 (6.0%)  2.24 (1.90)  I versus P = 0.31 [0.00, 0.62]  79 (5.5%)  2.40 (1.91)  I versus P = 0.36 [0.08, 0.64]  60 (4.2%)  2.03 (1.88)  I versus P = -0.07 [-0.39, 0.25]  81 (5.9%)  2.32 (2.07)  I versus P = 0.13 [-0.18, 0.44]  97 (6.8%)  2.73 (1.92)  I versus P = -0.02 [-0.31, 0.26] | 77 (5.4%)  3.46 (2.11)  D versus P = 0.24 [-0.08, 0.56]  92 (6.4%)  2.43 (1.87)  D versus P = 0.21 [-0.09, 0.51]  105 (7.3%)  2.65 (1.98)  D versus P = 0.23 [-0.04, 0.50]  72 (5.0%)  2.24 (2.24)  D versus P = -0.17 [-0.47, 0.14]  62 (4.6%)  2.37 (1.67)  D versus P = 0.12 [-0.21, 0.44]  65 (4.6%)  2.38 (1.80)  D versus P = 0.17 [-0.14, 0.48] | 78 (5.5%)  3.99 (2.31)  LS versus P = 1.22 [1.10, 1.34]  78 (5.5%)  2.82 (1.87)  LS versus P = 0.61 [0.49, 0.72]  115 (8.1%)  3.11 (2.03)  LS versus P = 0.80 [0.69-0.92]  92 (6.4%)  1.89 (1.96)  LS versus P = 0.03 [-0.08, 0.14]  82 (6.0%)  2.57 (1.76)  LS versus P = 0.48 [0.36, 0.59]  96 (6.7%)  2.69 (1.85)  LS versus P = 0.55 [0.44, 0.67] |

*Note. N,* Number of individuals; PRNS, Parent-rated negative symptoms; SDQ peer problems, Strengths and Difficulty Questionnaire peer problems subscale (self-rated); LS, Low-scoring group; I, Increasing group; D, Decreasing group; P, Persistent group. Cohen’s *d* measure of strength of association for unequal sample sizes (effect size of the standardized difference between the means; equal to the difference between the means divided by pooled *SD*); One randomly selected twin per pair included in analyses.

**Table S25**. *Correlations for psychotic experiences and negative symptoms with depression traits and SDQ emotional problems and other SDQ subscales*

|  | Paranoia | Hallucinations | Cognitive disorganization | Grandiosity | Anhedonia | PRNS |
| --- | --- | --- | --- | --- | --- | --- |
| SMFQ  SDQ emotional problems  SDQ conduct problems  SDQ hyperactivity  SDQ peer problems | .59 (<.001)  .46 (<.001)  .40 (<.001)  .32 (<.001)  .50 (<.001) | .48 (<.001)  .38 (<.001)  .35 (<.001)  .31 (<.001)  .27 (<.001) | .67 (<.001)  .62 (<.001)  .44 (<.001)  .61 (<.001)  .36 (<.001) | .04 (.11)  -.03 (.24)  .12 (<.001)  .06 (*p* = .03)  .04 (*p* = .18) | .17 (<.001)  .09 (.001)  .13 (<.001)  .06 (*p* = .02)  .24 (<.001) | .27 (<.001)  .25 (<.001)  .25 (<.001)  .20 (<.001)  .26 (<.001) |

*Note.* PRNS, Parent-rated negative symptoms; SMFQ, Short Mood and Feeling Questionnaire (self-rated) measure of depression traits; SDQ, Strengths and Difficulty Questionnaire emotional problems subscale, conduct problems subscale, hyperactivity subscale and peer problems subscale (self-rated); One randomly selected twin per pair included in analyses; Spearman’s rho correlation coefficients are reported owing to the skewed distributions of each variable. Pearson’s correlation coefficients are reported for SDQ and cognitive disorganization, and SDQ and anhedonia owing to the non-skewed distributions of either variable. *p* values in parentheses.

**Table S26**. *Descriptive statistics for depression traits and psychopathology subscales*

|  | SMFQ | SDQ Emotional problems | SDQ Conduct problems | SDQ Hyperactivity | SDQ Peer problems |
| --- | --- | --- | --- | --- | --- |
| *N* | 1,447 | 1,444 | 1,444 | 1,444 | 1,444 |
| Missing | 1 | 4 | 4 | 4 | 4 |
| Scale Range | 0-26 | 0-10 | 0-10 | 0-10 | 0-10 |
| Observed Mean | 4.83 | 3.16 | 1.84 | 3.87 | 1.87 |
| 95% CI | 4.55-5.12 | 3.03-3.28 | 1.76-1.92 | 3.74-4.00 | 1.77-1.96 |
| Median | 3 | 3 | 1 | 4 | 1 |
| Mode | 0 | 0 | 1 | 2 | 1 |
| *SD* | 5.49 | 2.45 | 1.60 | 2.51 | 1.78 |
| Variance | 30.15 | 6.00 | 2.57 | 6.30 | 3.16 |
| Skewness | 1.51 | 0.60 | 1.12 | 0.39 | 1.21 |
| Kurtosis | 1.89 | -0.36 | 1.33 | -0.55 | 1.57 |
| Cronbach’s α | .92 | .74 | .59 | .77 | .63 |

*Note*. SMFQ, Short Mood and Feelings Questionnaire; SDQ, Strengths and Difficulties Questionnaire.
